# Exploring the contribution of case study research to the evidence base for occupational therapy: a scoping review

**DOI:** 10.1186/s13643-023-02292-4

**Published:** 2023-07-31

**Authors:** Leona McQuaid, Katie Thomson, Katrina Bannigan

**Affiliations:** Glasgow Caledonian University, Glasgow, UK

**Keywords:** Case study research, Evidence-based practice, Occupational therapy, Single-case

## Abstract

**Background:**

Case study research is generating interest to evaluate complex interventions. However, it is not clear how this is being utilized by occupational therapists or how feasible it is to contribute to the evidence base. This scoping review explores case study research within occupational therapy in terms of how it is defined, the methodological characteristics adopted, such as data collection and analysis, and the range of practice contexts in which it is applied. We consider the viability of case study research for contributing to our evidence base.

**Methods:**

Opinion, text and empirical studies within an occupational therapy practice context were included. A three-step extensive search following Joanna Briggs Institute methodology was conducted in June 2020 and updated in July 2021 across ten databases, websites, peer-reviewed and grey literature from 2016 onwards. Study selection was completed by two independent reviewers. A data extraction table was developed and piloted and data charted to align with research questions. Data extraction was completed by one reviewer and a 10% sample cross checked by another.

**Results:**

Eighty-eight studies were included in the review consisting of (*n* = 84) empirical case study and (*n* = 4) non-empirical papers. Case study research has been conducted globally, with a range of populations across different settings. The majority were conducted in a community setting (*n *= 48/84; 57%) with populations experiencing neurodevelopmental disorder (*n* = 32/84; 38%), stroke (*n* = 14/84;17%) and non-diagnosis specific (*n *= 13/84; 15%). Methodologies adopted quantitative (*n *= 42/84; 50%), mixed methods (*n* = 22/84; 26%) and qualitative designs (*n* = 20/84; 24%). However, identifying the methodology and ‘case’ was a challenge due to methodological inconsistencies.

**Conclusions:**

Case study research is useful when large-scale inquiry is not appropriate; for cases of complexity, early intervention efficacy, theory testing or when small participant numbers are available. It appears a viable methodology to contribute to the evidence base for occupation and health as it has been used to evaluate interventions across a breadth of occupational therapy practice contexts. Viability could be enhanced through consistent conduct and reporting to allow pooling of case data. A conceptual model and description of case study research in occupational therapy is proposed to support this.

**Systematic review registration:**

Open Science Framework 10.17605/OSF.IO/PCFJ6.

**Supplementary Information:**

The online version contains supplementary material available at 10.1186/s13643-023-02292-4.

## Background

Developing evidence informed occupational therapy practice is a priority across international practice standards and research agendas [1, 2]. The challenge in achieving this, however, is multifaceted. Occupational therapists report a lack of research knowledge, time, resources and organizational support as barriers in the conduct of research [3–5]. Implementing findings from a research environment to the reality of clinical practice also presents a challenge despite knowledge translation and implementation strategies [6]. In practice, therapists use reasoning, experience and the client’s perspectives in addition to research [7, 8]. This holistic approach to service provision can be difficult to capture, but the need to demonstrate impact and quality outcomes remains.

Arguably, the challenge in evidencing the value of occupational therapy reflects the complexity of practice where the ‘the active ingredient’ is difficult to stipulate [9]. This is comparable to the ‘complexity turn’ of wider health and social care which acknowledges that interventions are not always linear processes with predictable outcomes [10]. In recognition of this, debate exists in occupational therapy about how best to develop the evidence base [11]. Whilst the need for large-scale inquiry and randomized controlled trials is evident, there is also a growing perception that this may not be appropriate to answer the full spectrum of practice-based questions [10]. Instead, the research method adopted should respond appropriately to the question being asked and often a range of methods may be necessary. In particular for occupational therapy, researchers should consider designs carefully, particularly when testing interventions, so the holistic nature of practice is not compromised [11]. A shift to a pluralistic approach which best serves the decision-making needs of practitioners may be more appropriate [12, 13].

Case study methodology—an in-depth analysis of a phenomenon within its real-world context [14]—has become increasingly popular in social sciences and is beginning to generate greater interest in occupational therapy [11, 15]. Focus on a single case in context presents a familiar and therefore potentially feasible approach to research for practitioners. As a methodology, it relies on the collection of multiple sources of data to gain an in-depth understanding of the case [14], resembling multiple sources of evidence informing decision making in practice [11]. Flyvberg [16] argues this detailed contextual knowledge is necessary for understanding human behaviours when there can be no absolutes. It therefore provides an alternative methodology where large-scale inquiry is not appropriate or feasible [14].

Confusion surrounds case study methodology in terms of how it is conducted, reported and consequently identified in the literature. Previous reviews have noted inconsistencies between methodology and design, mislabeling of case study research and a lack of clarity defining the case and context boundaries [15, 17]. It is often associated with qualitative origins, evolving from the natural and social sciences where disciplines such as anthropology, sociology and psychology demonstrate early application of the methodology and have since used it to grow their evidence base [18, 19]. However, case study research can be shaped by paradigm, study design and selection of methods, either qualitative, quantitative or mixed. Its flexibility as a methodology and variation in approach by seminal authors may add to the confusion. For instance, Stake [20] and Merriam [21] align to a qualitative approach whereas Yin [14] adopts more of a positivist approach with a priori design to examine causality. The language around case studies can also be synonymous with ‘non-research’ case reports, anecdotes about practice or educational case studies which do not include data collection or analysis [22]. However, case study methodology is research involving systematic processes of data collection with the ability to draw rigourous conclusions [17]. Hence, there is a need to better understand this methodology and bring clarity in defining it for research use in occupational therapy practice.

There are misconceptions that case study research can provide only descriptive or exploratory data and it is regarded as poorer evidence in the effectiveness evidence hierarchy [10]. However, in a meta-narrative review of case study approaches to evaluate complex interventions, Paparini et al. [15] noted diversity in epistemological and methodological approaches from narrative inquiry to the more quasi-experimental. As such, case study research offers flexibility to answer a range of questions aiding a pluralistic approach to research. Yin [14] suggests three purposes of case study research; (i) descriptive; describes a phenomenon such as an intervention; (ii) explorative; explores situations where there is no single outcome, and (iii) explanatory; seeks to explain casual relationships. Stake [20] on the other hand describes case study research as (i) intrinsic; to understand a single case, (ii) instrumental; where the case is of secondary interest to facilitate understanding to another context and (iii) collective; when multiple cases are studied around a similar concept. Whilst it has been criticized for lack of rigour and external validity [22], one case can be sufficient to make causal claims, similar to a single experiment [15]. A particular case can disprove a theory and prompt further investigation or testing [16]. Furthermore, Yin [14] reasons the accumulation of case studies may offer greater rigour, reliability and external validity of findings as a larger dataset is created. Through case replication and organized accessible storage, there is potential for data to be mined to conduct rigourous practice-based research [11, 23].

Some contention exists around the classification of single-case designs, including N-of-1 observational and experimental designs. Rice, Stein and Tomlin [24] argue the single-case experimental design (SCED) is not the same as a case study; however, Paparini [10] maintains this is coterminous with Yin’s explanatory case study aims. The International Collaborative Network of N-of-1 Trials and Single-Case Designs (ICN) articulates these designs broadly as the study of a single participant in a real-world clinical application [25]. This singular and contextual focus makes these designs appropriate to consider under the umbrella term case study research for the purposes of this review and exploring how N-of-1 may be a viable means to develop the occupational therapy evidence base.

Case study research has previously been advocated for in occupational therapy. Ottenbacher [26] originally described the small ‘N’ study as a tool for practitioners to address their responsibilities of documenting service provision effectiveness. Others have provided support for case study methodology to demonstrate clinical impact, overcome challenges of investigating complex phenomena and develop the occupational therapy evidence base [27–29]. It is presented as a good ‘fit’ for occupational therapy with untapped potential for contributing to the evidence base [11, 30]. Whilst these studies offer a justification for the use of case study research in occupational therapy and call for greater uptake of the method, no extensive review of empirical case study methodology in occupational therapy practice has been conducted. It therefore remains unclear if, and how, the methodology is being utilized, or how feasible it is to contribute to the evidence base. A scoping review was deemed the most appropriate methodology for this review as it has recognized value for researching broader topics [31]. It will identify all available, eligible evidence and chart key information from the literature to answer the research questions and identify any gaps in the knowledge base.

A preliminary search of PROSPERO, MEDLINE, the Open Science Framework and JBI Evidence Synthesis was conducted. A similar scoping review was published in 2020 but focused solely on the use of qualitative case studies in occupational therapy, therefore providing a restricted view of case study methodologies [32]. Equally, the literature search was conducted in 2017 and interest in this methodology has grown since; hence, there may have been a change in the use of qualitative case study research methods within occupational therapy in recent years.

This scoping review explores case study research within occupational therapy in terms of how it is defined, the methodological characteristics adopted, such as data collection and analysis, and the range of practice contexts in which it is applied. By reviewing case study research within the field, it will be possible to assess the viability of case study research for contributing to the evidence base for occupation and health. The enriched understanding of case study research within occupational therapy could identify areas for future research and strategies to improve evidence-based clinical outcomes for those accessing services.

## Review questions

This review aims to understand how case study research methodologies are used to contribute to the evidence base for occupational therapy practice. Specifically, it will identify and chart data to address the following sub-questions:

1. How is ‘case study’ defined as a research methodology in occupational therapy literature?
2. What are the methodological characteristics of case study research used in occupational therapy practice?
3. What are the contexts and recorded implications of case study research undertaken in occupational therapy practice?

## Methods

This scoping review was conducted in accordance with the Joanna Briggs Institute (JBI) methodology for scoping reviews [33] and, in line with best practice, used the updated Preferred Reporting Items Systematic Reviews and Meta-Analyses Extension for Scoping Reviews checklist (PRISMA-ScR)  (See Additional File 1 for PRISMA-ScR checklist) [34–36]. It was conducted in accordance with an a priori protocol [37], and any deviations from this are reported and justified.

### Inclusion criteria

#### Participants

This review considered studies where occupational therapy input is provided as the object of study or the ‘case’ within the case study; therefore, the inclusion criteria was not limited by participant characteristics. It is possible that included studies may not involve participants given the nature of case study research and non-empirical study types are also eligible for inclusion. This allowed the potential for a representative picture of who and what occupational therapists have studied using case study methodology.

#### Concept

Empirical studies using case study research methodology were included. Literature reviews, text or opinion pieces which discuss the value of case study research within occupational therapy practice were also included to ascertain how others have used or conceptualized the use of case study research to achieve evidence-based practice. Papers were excluded where a case study research design was not explicit, for example, a descriptive case report without data collection and analysis.

#### Context

Any area of occupational therapy practice was considered which spans health and social care, criminal justice, education and other diverse areas [38]. An a priori decision was made to exclude studies where the occupational therapy context could not be clearly defined, for example, multidisciplinary input or where practice was not the focus of the study, for example, describing an occupation only. All geographical locations were considered; however, as only articles written in English language were included, this may have created a geographical restriction through language limitations.

#### Types of sources

This scoping review included studies, as well as thesis and book chapters, if they involved empirical quantitative, qualitative and mixed method case study designs. Opinion, text or other articles which discuss the use of case study research in an occupational therapy practice context were also included. Case studies that are descriptive with no data collection and analysis were excluded. This was identified through reviewing the methods undertaken rather than how a study self-identified.

#### Search strategy

The search strategy aimed to locate both published and unpublished primary studies, reviews and text and opinion papers. To support the development and accuracy of the search strategy, a health systems librarian and occupational therapy profession specialist librarian were consulted in the early development stages. As per the JBI recommended three-step approach, an initial limited search of MEDLINE (EBSCO) and CINAHL (EBSCO) was undertaken to identify articles on the topic. The text words contained in the titles and abstracts of relevant articles, and the index terms used to describe the articles were used to develop a full search strategy. The scoping review process is iterative [33] so it was noted in the protocol that the search strategy may need to be adapted as the review evolved. As a result of the preliminary searches, a change was required through the addition of the search term ‘occupational science’. Without its inclusion, a valuable review on the use of case study research in occupational science which also included occupational therapy practice was missed [39]. Therefore, the addition of this term ensured a thorough search, recognizing the influence of occupational science on occupational therapy practice.

The search strategy, including all identified keywords and index terms, was adapted for each included information source and a second search was undertaken in June 2020 and updated on 7th July 2021. The full search strategies are provided in Additional file 2. The reference lists of articles included in the review were screened for additional papers plus a key author search to ensure all relevant studies were identified [33]. Studies published in English were included as the resources for translation were not available within the scope of this review.

The databases searched included MEDLINE (EBSCO), CINAHL (EBSCO), AMED (EBSCO), EMBASE (Ovid), PsychINFO (ProQuest) and Web Of Science. Sources of unpublished studies and grey literature searched included OpenGrey, Google and Google Scholar, OTDBASE, EthOS and OADT. To identify occupational therapy-specific literature, the content pages of practice publications Occupational Therapy News (UK), Occupational Therapy Now (Canada) and Occupational Therapy Practice (USA) were also screened from 2016.

Despite running preparatory searches, an unmanageable amount of papers were returned and on inspection many were dated in their approach to practice and language. For example, Pinkney [40] referred to ‘senile dementia’ and Pomeroy [41] referred to ‘handicap goals’. Therefore, to keep the review feasible as well as contemporary, a decision was made by the team to limit date parameters to 2016 onwards. This also meant that the OTSeeker database was omitted as a change from a priori as it has not remained comprehensive from this date due to lack of funding.

#### Study/source of evidence selection

Following the search, all identified records were collated and uploaded into Mendeley V1.19.4 (Mendeley Ltd., Elsevier, Netherlands) and duplicates removed. A decision was made not to use the JBI System for the Unified Management, Assessment and Review of Information (JBI SUMARI; Adelaide, Australia) as JBI SUMARI does not offer modifiable data extraction templates which was needed for this review [33]. Instead, studies were transferred to Rayyan QCRI (Qatar Computing Research Institute [Data Analytics], Doha, Qatar), a systematic review web application to manage the independent relevance checking process [42].

A screening tool was developed and piloted on a sample of studies by all three reviewers (LMQ; KT; KB) and adjusted until consensus reached to enhance clarity before continuing the full screening process. The screening tool served as a memory aid to ensure reviewers were being consistent in how the inclusion criteria was applied and all decisions were recorded on Rayyan QCRI. Titles and abstracts were screened by two independent reviewers for assessment against the inclusion criteria (LMQ; KT and KB reviewed half each). Due to the broad nature of the question and a lack of clarity in reporting case study research methodology in the title or abstract, where there was doubt, articles were included for full-text review to be as inclusive as possible. Potentially relevant papers were retrieved in full and assessed in detail against the inclusion criteria by two independent reviewers (LMQ; KT and KB reviewed half each). Full-text studies that did not meet the inclusion criteria were excluded, and reasons for their exclusion recorded. Any disagreements that arose between reviewers were resolved through discussion or with a third reviewer. Where required, the screening tool was refined following these discussions to create an audit trail and further enhance consistency in how inclusion criteria was applied in the screening process. Studies were not quality assessed, as per scoping review guidance [33], as the purpose of this scoping review was to map available existing evidence rather than consider methodological quality.

#### Data extraction

Data were extracted from papers using a data extraction tool developed by the reviewers into a Microsoft Excel spreadsheet (Redmond, Washington, USA). The tool was piloted by two independent reviewers initially on fourteen papers, an increase from the suggestion at protocol stage given the high number of included studies, and subsequently modified and revised. This clarified that only study designs stated, rather than conjected, would be extracted to reflect how authors self-categorize and define case study methodology. Additionally, it presented the need for a separate data extraction tool for non-empirical papers as some of the detail in the original tool was not relevant to review or discursive paper designs. The new tool captured details on reported strengths, limitations and explanations of data collection/analysis for the use of this methodology in occupational therapy practice. The updated data extraction tools are presented in Additional files 3 and 4.

Data extraction was completed by the first author and a 10% sample checked by a second reviewer. As recommended in the data extraction process [34], multiple reports from the same study were linked. The data extracted for empirical studies included specific details about the definitions, justification and citations of case study research, the methodological characteristics, the context in terms of practice setting and population and key findings and implications relevant to the review question [37]. Authors of papers were contacted to request missing or additional data, where required.

#### Data presentation

As specified in the protocol and recommended in the JBI scoping review guidance, the extracted data is presented in diagrammatic and tabular form. A narrative summary accompanies the charted results and describes how the results relate to the scoping review questions. A mapping approach to analysis was adopted as the objective of this scoping review was to collate the range of existing evidence and describe the methodological characteristics of case study research, rather than synthesis or appraise the evidence.

### Findings

**Fig. 1:**
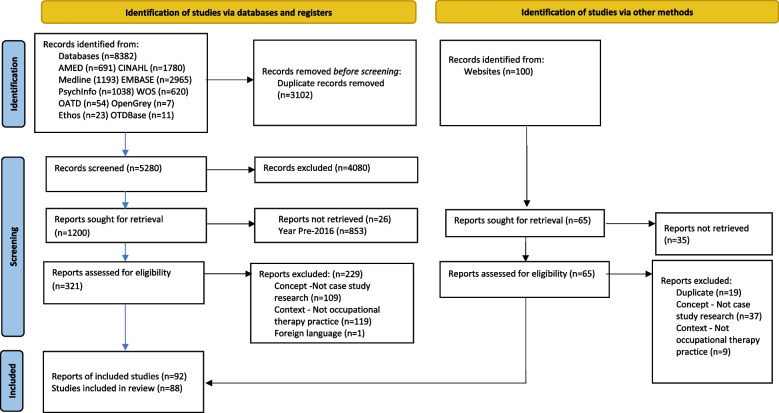
Search results and study selection and inclusion process [34]

In total, database and secondary searching returned 8382 studies (Fig. 1). After duplicates were removed, 5280 underwent title and abstract screening with 4080 articles excluded at this stage. Full-text screening and application of the updated 2016 date parameters led to a further 1108 articles excluded. This left 92 articles eligible for inclusion. This included seven reports linked to three studies which were subsequently combined [43] and four non-empirical papers consisting of a discussion piece and three literature reviews. Three of these reviewed the use of case study research in occupational therapy and/or occupational science prior to 2016, further justifying the decision to provide a more contemporary review. A final total of 88 records were included in the review; 84 empirical studies, and four non-empirical papers. The characteristics of included studies are presented in Additional files 5 and 6. The majority of studies were excluded due to not having an occupational therapy practice focus, for example, multidisciplinary or a description of the meaning of an occupation rather than in a practice context (see Additional File 7 for more detail).

After an initial dip from 2016, publication of empirical case study research shows a consistent trend from 2017 onwards; the lower number in 2021 is attributed to the search stopping mid-way through the year (July 2021) (Table 1). Across the 88 included studies, there is greater representation of the Global North with the USA (*n* = 24/88; 27%), Canada (*n* =12/88; 14%) and UK (*n* = 11/88; 13%) publishing the most case study research. Case study research has been adopted to address exploratory and explanatory aims, and as such, it has been used to understand the outcomes of interventions, to explore elements of practice such as theoretical models, and to understand occupation and occupational science concepts to inform practice. Empirical case study research was identified in journal articles (*n* = 77/88; 87%), predominantly in occupational therapy-specific journals (*n* = 56/88; 63%), theses (*n* = 6/88; 7%), abstracts (*n* = 4/88; 5%) and a book chapter (*n* = 1/88; 1%). The majority of case study research adopted a multiple case design (*n* = 64/84; 76%); however, single-case designs were also published (*n* = 19/84; 23%). Included studies have used multiple data collection methods including interviews, observation and outcome data and have been used in a range of practice settings across the life span. The empirical studies will now be mapped to answer each question of this review followed by mapping of the non-empirical studies.

**Table 1:** Summary of included studies

Study characteristics	Empirical (*N* = 84)	Non-empirical (*N* = 4)
**Publication year**		
2016	24	
2017	15	1
2018	11	1
2019	15	
2020	14	2
2021 (July)	5	
**Publication type**		
Journal article	73	4
Thesis	6	
Abstract	4	
Book chapter	1	
**Geographical context**		
USA	23	1
Canada	11	1
UK	10	1
Australia	8	1
Sweden	7	
Brazil	6	
Iran	5	
South Africa	4	
Ireland	1	
Korea	4	
Japan	2	
Portugal	2	
New Zealand	1	

### Mapping of empirical studies

1. How is ‘case study’ defined as a research methodology in occupational therapy literature?

**Fig. 2:**
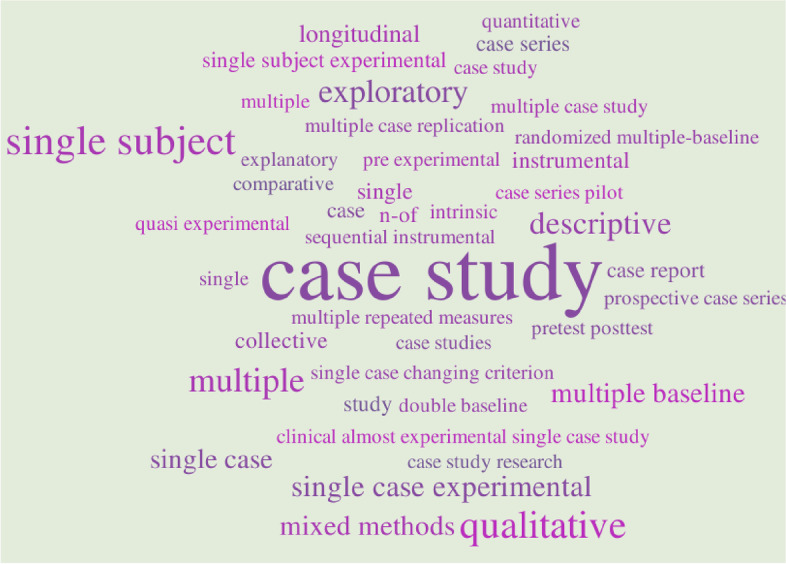
Phrase cloud illustration of study design as self-identified in included empirical studies. Size of the word illustrates frequency of use

There did not appear to be a consistent approach adopted across studies to define case study methodology. Figure 2 captures the various ways studies self-reported their methodological design (the more prominent the text, the more a word or phrase was featured in the data). Of the 84 empirical studies, 57% (*n* = 48/84) provided a definition or justification for the chosen case study research methodology. The most common cited explanations for adopting case study methodology were as follows: (i) to gain a deep understanding of the case (*n* = 28/84; 33%); (ii) to achieve this using multiple data sources, perspectives or baseline measures (*n* = 21/84; 25%) and (iii) to study the case in the real-world environment or context (*n* = 17/84; 20%). A need for comprehensive understanding was linked to the complexity of the case, such as a social interaction or human behaviour, e.g. Carrol [44] and Soeker & Pape [45]. Case study methodology was also justified as more suitable or practical when the phenomena was too complex or too little was already known for other data collection approaches, such as experiments or surveys to be used, e.g. Nilsson et al. [46] and Stickley & Hall [47]. Consequently, 10 studies specifically justified case study research as appropriate for early efficacy and feasibility studies, e.g. Peters et al. [48]. Case study methodology was described as a form of empirical enquiry or research by a small number of studies (*n* = 13/84; 15%), and in some instances, this was justified as being closely aligned to the principles of occupational therapy practice or a way to provide clinically relevant information, e.g. Kearns Murphy & Sheil [49] and Verikios et al. [50]. To a lesser extent (*n* = 6/84; 7%), case study methodology was described as a way to test theory.

Less than half of studies (*n* = 41/84; 48%) referred to seminal authors or included relevant case study methodological citations. Table 2 provides a summary of cited author explanation of case study research. Yin’s work was most commonly cited followed by Stake and Merriam whom were more associated, but not limited to, qualitative case studies. Dibsdall [51] and Hurst [52] justified their choice of Yin’s approach to case study methodology because it provided a clearer structure to follow.

2 What are the methodological characteristics of case study research used in occupational therapy practice?
  a Study design.

**Table 2:** Summary of cited author explanation of case study research

Author cited	Explanation of case study research
**Yin (**[14]**,p.15)**	Investigates a contemporary phenomenon (the ‘case’) in depth and within its real-world context, especially when the boundaries between phenomenon and context may not be clearly evident
**Stake (**[20]**, p. xi)**	Case study is the study of the particularity and complexity of a single case, coming to understand its activity within important circumstances
**Merriam (**[21]**, p.37)**	An in-depth description and analysis of a bounded system
**Flyvberg (**[16]**, p.241)**	The main strength of case studies is depth—detail, richness, completeness and within case variance. It is a necessary and sufficient method for certain research tasks in the social sciences
**Hamel, Dufour and Fortin (**[53]**, p.2)**	An in-depth study of the cases under consideration employing various methods
**Thorne (**[54]**, p.281)**	The case study or case in point is a fundamental component of knowledge development within an applied practice field
**Blatter and Haverland (**[55]**, p.19)**	A non-experimental research approach that differs from large-N studies in the following four characteristics; a small number of cases, a large number of empirical observations per case, a huge diversity of empirical observations and an intensive reflection on the relationship between concrete empirical observations and abstract theoretical concepts
**Ottenbacher (**[26]**, p.647)**	The single-system model of evaluation research provides a method for incorporating empirical procedures into clinical practice not available in traditional research methods
**Salminen et al. (**[29]**, p.3)**	Case study research seeks out rich, in-depth information. It aims to investigate a particular topic in its context from multiple viewpoints and it uses multiple methods and multiple data sources for its data collection. For occupational therapists, case study research offers a research approach that can be used to advance professional practice

Congruence between description of study design and the methods undertaken was not always consistent, and reporting of ethical approval to distinguish case study research from case reports was not always reliable. For example, two studies classified as case reports by the American Journal of Occupational Therapy [56, 57] include a methods section with data collection and analysis and have received ethical approval which would be more consistent with case study research methodology rather than a descriptive, non-research case report [14]. In contrast, Longpre et al. [58] documented that, after seeking guidance from three university review boards, ethics was not required for a case study approach despite including interview and document review data collection and an appropriate research citation.

b Methods of data collection

**Fig. 3:**
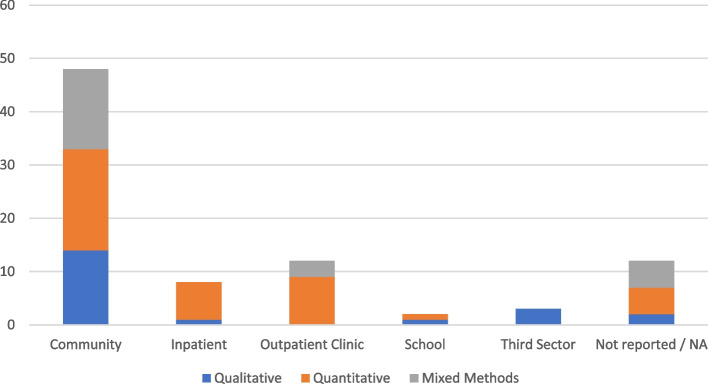
Number of studies per practice setting and data collection approach

Quantitative data collection methods accounted for the majority of methods (*n* = 42/84; 50%), but mixed methods (*n* = 22/84; 26%) and qualitative (*n* = 20/84; 24%) approaches were also used. As such, studies appeared to represent different research paradigms, although the authors positioning is only stated in two studies; critical realism [52] and constructivism [59]. Data collection methods varied dependent on practice setting with quantitative methods dominant in inpatient and outpatient settings whereas third sector only used qualitative methods (Fig. 3). Community settings used a mixture of quantitative, qualitative and mixed methods.

Quantitative data was used to evaluate effectiveness with testing pre and post intervention, and as such, they adopted explanatory, N-of-1, single-case experimental or observational designs. In contrast, qualitative designs were used in studies with an exploratory or descriptive purpose. Here, qualitative data added further understanding of the effects or acceptability of an intervention from a variety of perspectives. Data collection methods across qualitative studies included the use of semi-structured interviews, observation, document review, field diaries and focus groups. Observation was also evident in quantitative methods but for the purpose of gathering performance data and applying objective measures rather than descriptive or thematic purposes. Mixed methods case study research included a range of designs such as the single-case experimental design [60], multiple case study [61] and descriptive case study [62].

iii Outcome measures.

None of the included quantitative studies used exactly the same measures. However, the Canadian Occupational Performance Measure (COPM) was the most commonly used occupation-based outcome measure (*n* = 20/84; 23%) and to a lesser extent, the Assessment of Motor and Process Skills (AMPS) was used (*n* = 3/84; 4%). The Goal Attainment Scale (GAS) was also used (*n* = 5/84; 6%) and Kearns Murphy and Sheil [50] in particular advocated for its use in occupational therapy case study research, particularly in mental health settings. Non-occupation-specific measures of function were also used such as Range of Movement, Fugl-Meyer assessment, Sensory profiles and other condition-specific measures, e.g. Hospital Anxiety Depression Scale [63], Stroke Impact Scale [64] and Modified Checklist for Autism in Toddlers [65].

iv Methods of analysis.

Descriptive analysis and visual analysis to compare data graphed over time was used in quantitative experimental designs. Statistical analysis in the form of Rasch and frequency analysis was also employed in some instances [66–68] but this was largely in conjunction with visual analysis. Both Gustaffson et al. [69] and Gimeno et al. [70] suggested in their studies that visual analysis is preferable for single-case designs rather than statistical hypothesis testing due to the small number of participants. Thematic and content analyses were commonly used in qualitative studies in addition to descriptive statistics. For multiple case designs, within and cross case analysis was described [59, 64, 71–74]. Specifically, Yin’s approach to pattern matching [51, 73, 75, 76], explanation building [45] and matrix coding [77] was used. Two studies referred specifically to Stake’s approach to data analysis [59, 78].

e The case.

Few studies (*n* = 10/84; 11%) made the case explicit in terms of description, selection or boundaries. In particular, quantitative case study designs appeared not to define the case; therefore, the participant receiving occupational therapy was assumed to be the case. In these studies, the inclusion criteria, time and location of intervention appear to be the boundary. Alternatively, the provision of occupational therapy input as a process could be the case of interest. Fields [78] and Pretorious [79] exemplify a clearly defined case as an individual and both were bounded by the context of time and location. Haines et al. [78] and Hyett et al. [59] demonstrate a defined case as a process, occupational therapy provision and a social network respectively. Across the studies, the case, either stated or conjected, was predominately an individual (*n* = 72/84; 85%). Groups, namely families (*n* = 5/84; 6%) and organizations were also identified as the case (*n* = 4; 5%). The case was stated as a process in a small number of studies (*n* = 3/84; 4%); however, without a clear description of the case and boundary, it is challenging to accurately identify this within the included studies.

3. What are the contexts and recorded implications of case study research undertaken in occupational therapy practice?
  a Practice contexts

Occupational therapy case study research were conducted with various client groups across a range of practice settings (Additional files 8 and 9). The majority were based in the community (*n* = 48/84; 57%); however, the practice context or setting where the research was carried out was not always clearly reported (*n* = 11/84; 13%). Interventions adopting therapeutic use of occupation and activity were apparent, such as feeding [80], gaming [81, 82], gardening [83] and play [84–86]. This was more prevalent in outpatient or community settings with inpatient settings adopting more of a compensatory approach [87] to facilitate engagement in occupations as an end, rather than the therapeutic use of occupation itself as a means. Across all practice settings, the most common occupational therapy interventions were sensory-based interventions (*n* = 10/84; 12%) for example Giencke Kimball et al. [88], Go & Lee [89] Hejazi-Shirmard et al. [90], and provision of assistive equipment (*n* = 9/88; 11%) for example Cruz et al. [91], Golisz et al. [92] and Teixeira & Alves [93]. In other instances (*n* = 4/84; 5%), provision of occupational therapy was described as the intervention, subsequently involving a range of input rather than a single defined intervention, for example Kearns Murphy & Sheil [49], Haines et al. [78] and Pretorius [79].

Although all studies had a practice focus, not all were intervention specific but investigated a broader aspect of practice and so did not always include participants (*n* = 11/84; 13%). For example, Carey et al. [94] conducted an instrumental case study on the case of occupational therapy practice in the broad context of mental health services in Saskatchewan, Canada. This involved reviewing documentation and records from practice rather than including a population group or specific intervention. Others focused on particular assessments used in practice [95, 96] using conceptual frameworks in practice [52, 59] and practice at the organization or community level [47, 71, 97, 98].

For studies that included a population group, case study methodology was used across the life span; adults (*n* = 27/84; 32%) children (*n *= 24/84; 29%) and to a lesser extent, older adults (*n* = 6/84; 7%). It was also used with mixed age populations (*n* = 21/84; 25%) for instance, with families. Across all age groups, case study research was conducted largely with populations experiencing neurodevelopmental disorder (*n* = 32/84; 38%), stroke (*n* = 14/84; 17%) and ill mental health (*n* = 9/84; 11%) but was not always diagnosis specific (*n* = 13/84; 15%) (Additional file 9). For example, in Dibsdall’s [51] case study of a reablement service, occupational therapists provided a service to individuals with a range of diagnoses. Similarly, Fischl et al. [72] supported older adults with digital technology-mediated occupations irrelevant to a particular diagnosis.

b Recorded implications for practice.

As the majority of studies had an intervention focus (*n* = 73/84; 87%), they were able to draw conclusions in terms of how and why an intervention works. However, implications for practice in terms of intervention efficacy were often presented as preliminary or pilot with recommendations for further research including larger sample size studies. Through multiple data collection methods, some studies incorporated participant, family or therapist views to triangulate data and draw conclusions about the acceptability of an intervention [50, 62, 99]. As an example, Peny-Dahlstand et al. [99] includes a clear diagram illustrating how multiple data sources are collected from the patient, the therapist and the organizational perspective to analyse feasibility in terms of acceptability, efficacy, adaptation and expansion. Details of the Cognitive Orientation to daily Occupational Performance intervention are aligned to a protocol giving the reader a sense of how this can be implemented in practice. Similarly, a detailed description of the intervention, case and/or context can aid transferability [14] as in Carlsedt et al.’s [64] overview of the BUS TRIPS intervention.

The remaining studies (*n* = 11/84; 13%) added to the understanding of non-intervention aspects of practice such as the use of models, frameworks and assessment tools within the practice context or recommended policy changes. For example, Soeker and Pape [45] explored the experiences of individuals with a brain injury of the Model of Self-Efficacy (MOOSE) as it was used by occupational therapists to support their return to work journey. Using an exploratory multiple case design, the authors were able to conclude that the MOOSE is a useful model in this area of practice as well as increasing understanding of how and why it supported work retraining.

### Mapping of non-empirical papers

Four non-empirical papers that reviewed the use of case study research related to occupational therapy were included in this review. These were integrative reviews of case study research in occupational therapy [100], occupational science [39] and a scoping review of qualitative case study research [32] together with a discussion of the applicability of single-case experimental designs to occupational therapy [101]. The literature review searches were conducted in either 2016 or 2017 and identified 32 [100], 27 [32] and 18 studies [39]. Results reflect the findings of the empirical studies in the current review, suggesting a global uptake of case study research in occupational therapy across a diversity of practice settings used to understand interventions as well as broader concepts related to practice.

Together, the reviews present the defining features of case study methodology as investigating a phenomenon (i) in depth, (ii) in its real-life natural context, and (iii) using multiple sources of data for triangulation. Jonasdittor et al. [39] and Carey [100] both suggest case study methodology can cross research paradigms and therefore can be qualitative, quantitative or mixed methods in nature. Lane [101] somewhat contradicts this stating that case studies are a form of descriptive qualitative inquiry and therefore described the quantitative single-case experimental design (SCED) as distinct and separate from case study research. However, Lane [101] also acknowledged that multiple sources of data may be used including narrative records but this should be considered secondary to observing trends in data because the primary focus is to determine the effect of the intervention. In the SCED, multiple data collection points are used for in-depth understanding to measure change and make appropriate intervention responses. Hercegovac et al. [32] did not make a distinction about data collection methods but sought only qualitative case study research. Reflective of this, the majority of studies identified by Jonasdottir et al.’s [39] and Hercegovac et al.’s [32] reviews were qualitative but in Carey’s review [100] they were mixed methods. Quantitative studies were less common.

All four papers comment that generalizations cannot be made from a single case. Instead, providing a thick description of characteristics and information about the case was deemed necessary to help the reader understand the context and determine transferability of the case. Collecting and comparing across cases was also noted to provide greater validity [101]. Despite this, Hercegovac et al. [32] identified only 18% of studies that had adequately defined the case. All review and discussion papers conclude that case study or single-case experimental designs are appropriate in the study of occupation and health. They support the wider adoption of this methodology to advance the occupational therapy evidence base because it offers a rigourous but flexible approach to study complexity in the real-world practice environment. It is presented as a ‘familiar, appropriate tool’ ([100]; p.1293) to develop evidence informed practice.

**Fig. 4:**
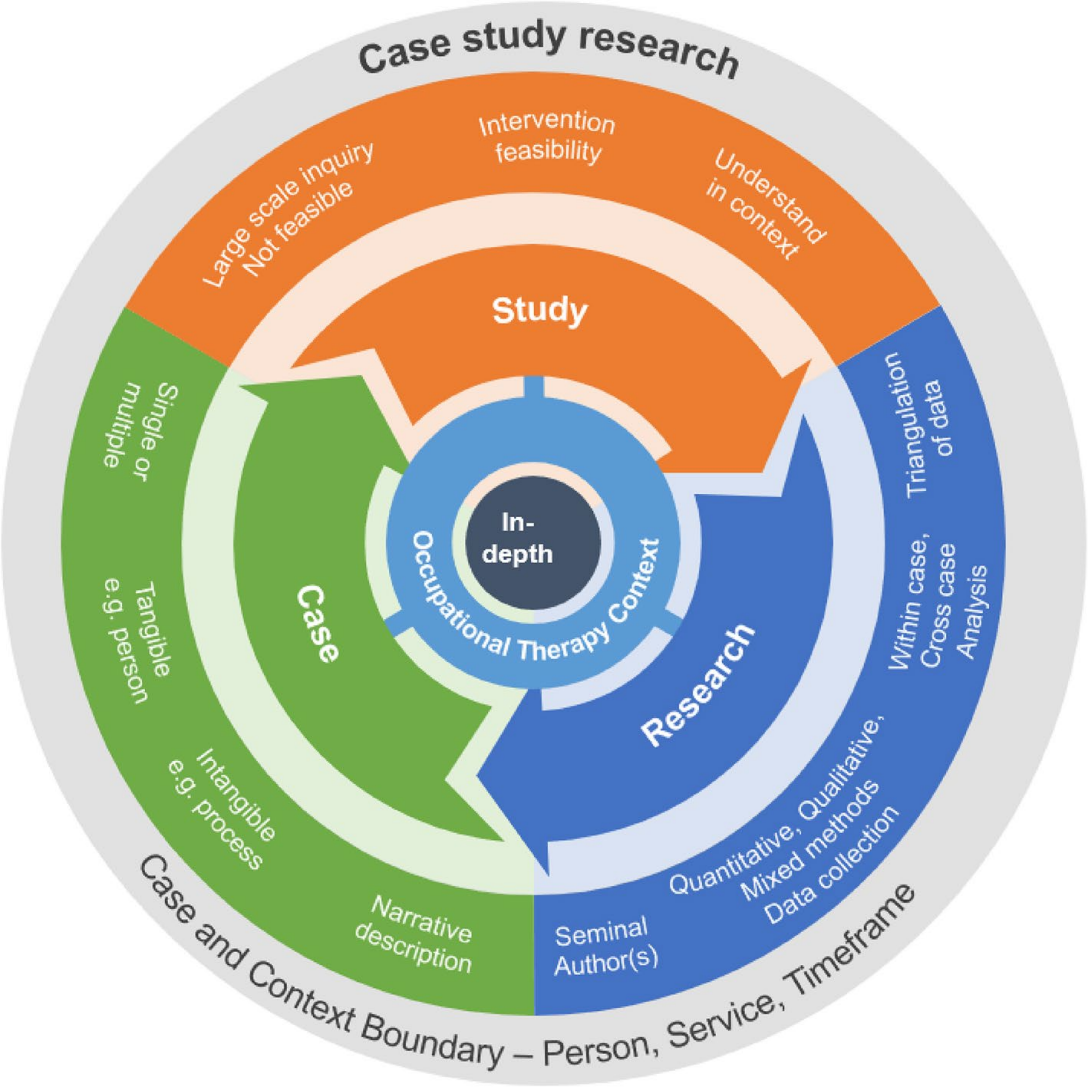
Proposed conceptual model describing case study research in occupational therapy practice

The findings of this review, in conjunction with the wider literature knowledge base, are integrated in Fig. 4 as a proposed conceptual model to illustrate how case study research can be applied in occupational therapy practice. It highlights the three important elements of the methodology as the ‘Case’ of interest, the rationale for the ‘Study’ design and that it is a ‘Research’ method. Central to the application of this methodology is the aim to achieve an in-depth understanding of a phenomenon within the occupational therapy practice context. To compliment Fig. 4, a description of case study research within occupational therapy is proposed as;

‘a flexible methodology that can cross research paradigms where the focus is to gain an in-depth understanding of a case in the real-life practice context. The case and context can reflect any aspect of occupational therapy, but must be clearly defined and described within a given boundary. A comprehensive understanding of the case or cases should be gained through triangulation of data collection either through multiple data sources or multiple time points.’

## Discussion

This scoping review explored the use of case study research within the occupational therapy evidence base from 2016 to 2021. A large number of studies (*N* = 88) were identified across a variety of practice settings and following a dip after 2016, publication trends appeared consistent over this period. This suggests that case study research has potential viability for contributing to the evidence base of occupation and health. However, the findings of this review identified inconsistencies in how case study research was defined and variation in the methodologies adopted. Therefore, to maximize its potential as an evidence building tool, further clarity on case study methodology is needed. It is hoped that this review, in particular the proposed definition and conceptual model, will help achieve this.

A key issue highlighted was the lack of consistent or easily identifiable terms used to describe the methodology. Some studies defined the design by number of cases (e.g. single/multiple), by purpose (e.g. exploratory, descriptive, experimental) or by data collection (e.g. quantitative, qualitative, mixed). Other terms were also used such as ‘almost experimental’, ‘case series’, ‘changing criterion’ and ‘case report’. Hyett [17] suggested case study, as a research approach, has been confused with the non-research-based case report and this is supported by the findings of the current review. Self-identified ‘case studies’ were excluded, in line with the inclusion criteria, if they did not report data collection or analysis. In addition, journal classification of study type was at times incongruent with the methodology taken, e.g. Proffitt et al. [57]. Alpi & Evans [102] highlight this lack of distinction not only in journal classification but also in database indexing. They propose that case study is a rigourous qualitative research methodology and case report is a patient or event description. Based on this, the Journal of Medical Library Association updated classification of descriptive manuscripts previously known as case studies to case reports and case studies as a research methodology are now identified as original investigations. Despite this effort at clarification, there is still room for debate. Where Alpi & Evans [102] suggest N-of-1 single subject studies fit the case report label, Paparini et al. [10] aligns this to the explanatory case study. Therefore, this review adopted Yin’s [14] term ‘case study research’ as a common language that can be used by occupational therapists in the conduct and reporting of this methodology. It is suggested this will make the distinction clear from case report or non-research.

The issues highlighted in this review reflect current debate about case study research methodology. A key issue identified with empirical case study research was the inadequate description of the case and boundary so that it could be easily identified by the reader. Other reviews of case study research in occupational therapy included in this review [32, 39, 100] also identified this as a concern pre-2016 and Hyett [17] identified this more broadly in the literature, but particularly a concern for health and social science case studies. A clearly identifiable case, with detailed description including the boundary and context, is necessary for practitioners to understand how it may translate to their own practice. A case is not synonymous with participant and, whilst it can be an individual of interest, it can also take a more intangible form of a process such as intervention delivery, practice networks or other practice areas of interest such as theory.

As a form of inquiry, case study research provides context-specific, practice-based evidence, so the practice context must be understood. This in-depth, contextual understanding provides an alternative to studies seeking breadth of knowledge or generalizations and is thus the unique characteristic of case study research [11]. For this reason, ‘in-depth’ inquiry and ‘occupational therapy practice context’ are positioned at the core of the proposed descriptive model, encapsulated by the ‘case and context boundary’ as essential elements to case study research methodology (Fig. 4).

Case study research has been shown to be a flexible methodology both in design and purpose. Of particular interest to evidence building is its use to explore the efficacy and feasibility of an intervention in the real-life practice context. These findings support the assertions of previous authors who have suggested that case study research can be used to demonstrate clinical impact of interventions and to investigate complex multifactorial phenomena [11, 27–29]. Particularly in areas of innovative or emerging practice, case study research can provide a way to capture impact when participant numbers or resources are not available to conduct larger-scale inquiry. Stickley and Hall [47], for instance, specifically state that their study is the first known investigation into social enterprise in occupational therapy. As a first step to building evidence, a descriptive or single-case account can therefore provide an important grounding on which to build upon. The need for timely evidence during the Covid-19 pandemic demonstrated an acute awareness of this but it has also been recognized as a process of cumulative evidence building in occupational science [103] and more broadly across other disciplines [104]. Of note however is Flyvbjerg’s [16] argument that the case study holds value beyond pilot or preliminary data. Whilst it may be difficult to generalize from a case study, particularly in terms of process, the outcomes can contribute to knowledge when used to test a theory or data pooled across cases.

By mapping the findings of this review, case study research appears to mirror the broad and varying nature of occupational therapy. It reflects occupational therapy as a direct service provided to individuals or groups, but also to others on a client’s behalf [105]. Organization, population and system-level practice is also recognized as an important aspect of occupational therapy practice [38] and was reflected in the included cases [71, 97]. Case study research therefore not only has the potential to evidence impact through intervention outcomes, but also has wider health and well-being impact potential by exploring and advocating for occupational therapy across the full spectrum of practice including diverse areas.

Occupational therapy was provided in a range of settings including hospital, community and industry sectors. Interventions adopted illustrate the global variation in occupational therapy practice. For instance, compression bandaging [69, 106] and electrical stimulation [107, 108] are not aspects of standard practice in the UK but reflect other international practice standards [109, 110]. Interventions were wide ranging and reflective of those described in the American Occupational Therapy Process and Domain Framework [38]. This included therapeutic use of occupation [83], interventions to support occupation [111], education and training-based [112], advocacy-based [76], group-based [113] and virtual interventions [114]. Narrowing the intervention to a single entity was not always possible or appropriate reflecting the complexity of occupational therapy practice and several authors, for example Kearns Murphy & Sheil [49] and Pretorious [79] instead reported occupational therapy as the intervention involving a range of activities and approaches that were meaningful and goal directed for the client.

A suggested strength of case study research identified by the findings is the similarity between the research process and clinical practice. Fleming [115] had suggested that practitioners generate hypothesis in clinical practice to test theory and problem solve elements of the therapy process for example, why an intervention may not be working as expected. Similarly, case study research has been used to test theory in evaluative or explanatory designs. Methods of data collection (e.g. observation, outcome measurement, document review, interview, client feedback) and analysis (e.g. descriptive, visual, pattern-matching outcomes) bear resemblance to how evidence is collected in practice to inform the intervention process [116]. The term ‘pattern matching’ is an analytic strategy adopted by Yin [14] in case study research to compare patterns in collected data to theory. However, pattern matching is also evident in occupational therapy clinical reasoning literature, particularly in relation to how practitioners utilize tacit knowledge to inform decision making [117, 118]. This insight into case study research supports the perspective that it may be a more familiar and therefore achievable approach to evidence building for practitioners.

The challenge of capturing the complexity of practice has previously been cited as a barrier to research engagement and evidence-based practice in occupational therapy [11]. In contrast to this, case study research was largely justified as the chosen methodology because it allowed for individual tailoring of the intervention to the case and context [72, 74, 75]. The ability to provide a narrative description of the case, context, intervention and how it was implemented or adapted was seen across case study research, including single-case experimental designs (SCED). This idea of ‘individualization’ of treatment is also noted by Fleming [119] to differentiate occupational therapy clinical reasoning from medical procedural reasoning. The effectiveness of occupational therapy is not solely based on a prescriptive treatment, but is also influenced by the interactions between the therapist and service user and the particulars of that context. Therefore, if thinking on clinical reasoning has evolved to capture the important nuances of interactive reasoning [115] and furthermore embodied practice [118] then it would seem appropriate that the research approach to building evidence should also. A pluralistic approach whereby there is a valued position for both case study research and larger-scale inquiry to capture both the depth and breadth of practice would seem fitting. Collecting and pooling case study research data from practice can capture these important elements and allow for pattern matching or synthesis. In this way, case study research can hold value for evidence building, just as the randomized controlled trial, or other larger-scale inquiry, does for generalizability with the potential to inform policy and practice.

Based on the findings from this review, collecting case studies from practice to develop an evidence base is potentially viable given its uptake across practice areas and relatively consistent publication. In psychotherapy, Fishman [23] advocated for a database of cases which follow a systematic structure so they can be easily understood, recognized and data compared. Journals dedicated to publishing case data using a methodical format have since evolved in psychotherapy [120]. In occupational therapy, the Japanese Association of Occupational Therapists [121] collects practical case reports from members using dedicated computer software to host a collective description of occupational therapy practice. There is potential then to adopt this even on an international basis, where occupational therapy practice can be shared and measured. The challenge however is in achieving a systematic approach to how case study research data is collected and recorded to allow for meaningful comparisons and conclusions to be drawn.

In this review, quantitative and mixed method designs used a range of different outcome measures which is not conducive to pooling cross case data. Goal Attainment Scaling (GAS) is an outcome measure that defines individualized goals and relative outcomes to determine therapeutic effectiveness [122]. It is a measure advocated for its applicability across areas of practice but also for research, both large-scale inquiry and case study research [123]. In this review, it was used across age groups, in the community, outpatient settings and schools and in the areas of neurodevelopmental disorder, stroke, brain injury and ill mental health.

Kearns-Murphy & Sheil [49, 123, 124] adopted Goal Attainment Scaling in their longitudinal case study and explored the different methods of analysis of the measure. They concluded that charting GAS scores at multiple timepoints is beneficial to case study research as it adds to the ‘in-depth’ analysis providing insight into the fluctuations of therapy and outcomes in the real-life context. Visual analysis of charted scores is then an appropriate analytic technique for intervention-based case study research. Two time points, before and after, are more suited to large-scale inquiry for generalization but in the case study, only the performance of an individual on a particular day is highlighted which may be influenced by several contextual factors. Given these assertions, adopting a consistent outcome measure across practice such as GAS, would allow for in-depth, case and context-specific understanding that could also be comparable and pooled across cases.

### Strengths and limitations of the scoping review

This review searched published and grey literature using a variety of terms that have been used interchangeably with case study research with the aim of conducting a comprehensive overview. It followed a peer-reviewed protocol with systematic and transparent processes. JBI methodology for the conduct of scoping reviews was followed and bibliographic software (Mendeley) and systematic review software (Rayyan) was used to manage citations and the screening process. Additionally, an updated search was completed in July 2021 to enhance the timeliness and relevance of findings.

Ten databases were searched and no further relevant articles were identified through websites or citation searching, affirming that a thorough search had been conducted. However, to balance a comprehensive search with the practicality of resources, some decisions were made which may impact the inclusivity of the review. Western dominant databases and English language limits were applied because of translation resource availability within the research team. The search algorithm was developed and tested with an academic health librarian at the protocol stage; however, as case study methodology was not always clear from the title and abstract, an unmanageable amount of data was presented at full-text stage. To manage the number of records, inclusion criteria was changed to provide a contemporary overview from 2016 rather than 1990. This may introduce some bias to the review, where relevant articles pre-2016 or in other languages were omitted. However, the narrower focus allowed for in-depth data mapping to maximize the value of findings for informing future practice and research. Without taking this step, the output would likely have been more superficial. As a large number of 88 studies were still included in total, it was felt an appropriate balance had been achieved.

## Conclusions

Findings suggest that case study research is a viable methodology to contribute to the evidence base for occupation and health as it has been used to evaluate interventions across a range of occupational therapy practice contexts. It has been used for cases of complexity, early intervention efficacy and feasibility, theory testing or when small participant numbers are available, in other words, when large-scale inquiry is not appropriate.

Inconsistencies were identified that mirror findings of case study research methodology in other disciplines. In particular, case study design and description of the case and boundary were poorly reported. Therefore, this review proposes that a common language is used—case study research—to define this flexible methodology. A description and conceptual model are proposed to assist in clarifying how case study research can be applied and reported in occupational therapy. Consistent reporting as a research form of inquiry improved description of the case and boundary and reference to seminal authors would help differentiate research from non-research cases and enhance viability for pooling cases together through more consistent, systematic conduct and reporting.

### Implications for research and practice

There is a need to distinguish case study as a research method, separate from the illustrative case report and from purely qualitative inquiry, for it to be identifiable in the literature to reduce confusion and capability concerns. Therefore, the term ‘case study research’ is proposed when referring to the research methodology specifically. Citation of seminal authors alongside this description of study design would aid visibility of case study research as distinct from non-research and could also support appropriate journal classification. Greater clarity in reporting case description, including a narrative summary of the case, context and boundary of study is also an area for development. The development of a systematic template for the collection and reporting of case study data, ideally mirrored internationally, would likely be an ideal solution. This would potentially build capability for the conduct of rigourous case study research, help make it more identifiable in the literature and support pooling data across studies for synthesis and generalization, thereby overcoming the criticisms of case study research. Through accurate and detailed description of case context and boundary, practitioners would more easily be able to identify if the information is relevant to their own practice context.

Case study research has been shown to be appropriate for use across settings and populations, therefore pooling data could enable services to benchmark. Practitioners seeking to explore research within their practice are encouraged to consider the case study approach for its flexible nature and suitability to the person-centred values of occupational therapy. Use of a consistent outcome measure would support pooling of data and, as GAS is specific to the individual rather than practice setting, services may want to explore it as a measure suitable for intervention-based case study research.

## Supplementary Information

**Additional file 1.** Preferred Reporting Items for Systematic reviews and Meta-Analyses extension for Scoping Reviews (PRISMA-ScR) Checklist.

**Additional file 2.** Search strategy.

**Additional file 3.** Data extraction instrument (Empirical studies).

**Additional file 4.** Data extraction instrument (Non-empirical studies).

**Additional file 5.** Characteristics of included empirical studies.

**Additional file 6.** Characteristics of included non-empirical papers.

**Additional file 7.** Studies ineligible following full-text review post 2016.

**Additional file 8.** Heat map contrasting interventions and practice contexts. Numbers and shading represent number of studies.

**Additional file 9.** Heat map contrasting population characteristics of age and diagnosis categories. Numbers and shading represent number of studies.

## Data Availability

The datasets generated and analysed during the current study are available in the UK Data Service ReShare repository, [10.5255/UKDA-SN-855706].

## Abbreviation

AMPS: Assessment of Motor and Processing Skills
COPM: Canadian Occupation Performance Model
GAS: Goal Attainment Scaling
ICN: International Collaborative Network of N-of-1 Trials and Single-Case Designs
JBI: Joanna Briggs Institute
MOOSE: Model of Self-Efficacy
SCED: Single-case experimental design

## References

[1] World Federation of Occupational Therapists (2017). International occupational therapy research priorities. OTJR: Occup Participation Health.

[2] Royal College of Occupational Therapists. Identifying research priorities for occupational therapy in the UK What matters most to the people accessing and delivering services?. 2021. www.rcot.co.uk/publications Accessed 11 Feb 2022.

[3] Thomas A, Law M (2013). Research utilization and evidence-based practice in occupational therapy: a scoping study. Am J Occup Ther.

[4] Samuelsson K, Wressle E (2015). Turning evidence into practice: barriers to research use among occupational therapists. Br J Occup Ther.

[5] di Bona L, Wenborn J, Field B, Hynes SM, Ledgerd R, Mountain G (2017). Enablers and challenges to occupational therapists’ research engagement: a qualitative study. Br J Occup Ther.

[6] Perkins B, di Tommaso A, Molineux M, Power P, Young A (2020). Knowledge translation approaches in occupational therapy: a scoping review. J Occup Ther Educ.

[7] World Federation of Occupational Therapists. Guiding principles for the use of evidence in occupational therapy. 2021. https://wfot.org/resources/guiding-principles-for-the-use-of-evidence-in-occupational-therapy Accessed 15 Jan 2022.

[8] Sackett DL, Rosenberg WMC, Gray JAM, Haynes RB, Richardson WS (1996). Evidence based medicine: what it is and what it isn’t. BMJ.

[9] Creek J (2003). Occupational Therapy defined as a complex intervention.

[10] Paparini S, Green J, Papoutsi C, Murdoch J, Petticrew M, Greenhalgh T (2020). Case study research for better evaluations of complex interventions: rationale and challenges. BMC Med.

[11] McQuaid L, Thomson K, Bannigan K (2022). Case Study Research: Building the occupational therapy evidence base one case at time. Scan. J. Occup..

[12] Greenhalgh T, Papoutsi C (2018). Studying complexity in health services research: desperately seeking an overdue paradigm shift. BMC Med.

[13] Tomlin G, Borgetto B (2011). Research pyramid: a new evidence-based practice model for occupational therapy. Am J Occup Ther.

[14] Yin RK (2018). Case study research and applications: design and methods.

[15] Paparini S, Papoutsi C, Murdoch J, Green J, Petticrew M, Greenhalgh T (2021). Evaluating complex interventions in context: systematic, meta-narrative review of case study approaches. BMC Med Res Methodol.

[16] Flyvbjerg B (2006). Five misunderstandings about case-study research. Qual Inq.

[17] Hyett N, Kenny A, Dickson-Swift V (2014). Methodology or method? A critical review of qualitative case study reports. Int J Qual Stud Health Well-being.

[18] McLeod J (2010). Case study research in counselling and psychotherapy.

[19] Harrison H, Birks M, Franklin R, Mills J (2017). Case study research: foundations and methodological orientations. Forum: Qual Soc Res.

[20] Stake RE (1995). The art of case study research.

[21] Merriam SB (1988). Case study research in education: a qualitative approach.

[22] Yazan B (2015). Three approaches to case study methods in education: Yin, Merriam, and Stake. Qual Rep.

[23] Fishman DB (2001). From single case to database: a new method for enhancing psychotherapy, forensic, and other psychological practice. Appl Prev Psychol.

[24] Rice MS, Stein F, Tomlin G (2019). Single-case experimental design. Clinical research in occupational therapy.

[25] Nikles J, Onghena P, Vlaeyen JWS, Wicksell RK, Simons LE, McGree JM (2021). Establishment of an international collaborative network for N-of-1 trials and single-case designs. Contemp Clin Trials Commun.

[26] Ottenbacher KJ (1986). Evaluating clinical change: Strategies for occupational and physical therapists.

[27] Colborn AP (1996). A case for case study research. Am J Occup Ther.

[28] Fisher I, Ziviani J (2004). Explanatory case studies: implications and applications for clinical research. Aust Occup Ther J.

[29] Salminen A, Harra T, Lautamo T (2006). Conducting case study research in occupational therapy. Aust Occup Ther J.

[30] Sonday A, Ramugondo E, Kathard H (2020). Case study and narrative inquiry as merged methodologies: a critical narrative perspective. Int J Qual Methods.

[31] Peters MDJ, Godfrey CM, Khalil H, McInerney P, Parker D, Soares CB (2015). Guidance for conducting systematic scoping reviews. Int J Evid Based Healthc.

[32] Hercegovac S, Kernot J, Stanley M (2020). How qualitative case study methodology informs occupational therapy practice: a scoping review. OTJR Occup Participation Health.

[33] Peters MDJ, Godfrey C, McInerney P, Munn Z, Tricco AC, Khalil H. Chapter 11: Scoping Reviews (2020 version). In: Aromataris E, Munn Z (Editors). JBI Manual for Evidence Synthesis, JBI 2020. https://synthesismanual.jbi.global. Accessed 23 Oct 2021.

[34] Page M, McKenzie J, Bossuyt P, Boutron I, Hoffmann T, Mulrow C (2021). The PRISMA 2020 statement: an updated guideline for reporting systematic reviews. BMJ.

[35] Peters MDJ, Godfrey C, Mcinerney P, Khalil H, Larsen P, Marnie C (2022). PROOF Best practice guidance and reporting items for the development of scoping review protocols and 11 knowledge translation program. JBI Evid Synth.

[36] Tricco AC, Lillie E, Zarin W, O’Brien KK, Colquhoun H, Levac D (2018). PRISMA extension for scoping reviews (PRISMA-ScR): checklist and explanation. Ann Intern Med.

[37] McQuaid TL, Bannigan K. Exploring the contribution of case study research to the evidence base for occupational therapy: a scoping review protocol. JBI evid Synth. 2021;19(8):2040-7. 10.11124/JBIES-20-00192 34400600

[38] American Occupational Therapy Association (2020). Occupational therapy practice framework: domain and process 4th edition. American journal of occupational therapy. Am Occup Ther Assoc.

[39] Jónasdóttir SK, Hand C, Misener L, Polgar J (2018). Applying case study methodology to occupational science research. J Occup Sci.

[40] Pinkney L (1997). A comparison of the Snoezelen environment and a music relaxation group on the mood and behaviour of patients with senile dementia. Br J Occup Ther.

[41] Pomeroy VM, Conroy MC (1997). Setting handicap goals with elderly people: a pilot study of the Life Strengths Interview. Clin Rehabil.

[42] Ouzzani M, Hammady H, Fedorowicz Z, Elmagarmid A (2016). Rayyan-a web and mobile app for systematic reviews. Syst Rev.

[43] Lefebvre C Glanville J, Briscoe S, Featherstone R, Littlewood A, Marshall C, Metzendorf M-I, noel-Storr A, Paynter R Rader, T, Thomas J, Wieland LS. Chapter 4: Searching for and selecting studies. In: Higgins JPT, Thomas J, Chandler J, Cumpston M. Li T, Page MJ, Welch VA, (editors). Cochrane Handbook for Systematic Reviews of Interventions version 6.3 (updated February 2022). Cochrane; 2022 Available from: https://training.cochrane.org/handbook/current/chapter-04

[44] Carroll AM. Caregiving teams and toddlers study: two single-case changing criterion designs to examine the effects of a two parent-mediated intervention for families with toddlers at risk or with autism spectrum disorder. Dissertation abstracts international section a: Humanities and social sciences. The University of North Carolina at Chapel Hill; 2020.

[45] Soeker MS, Pape C (2019). The use of the model of occupational self-efficacy for work retraining: a multiple case study. Occup Ther Int.

[46] Nilsson A, Johansson U, Ekbladh E, Bernspång B, Hellman T, Eriksson G (2020). Work potential and work performance during the first try-out of the person-centred return to work rehabilitation programme rework-stroke: a case study. Healthcare.

[47] Stickley AJ, Hall KJ (2017). Social enterprise: a model of recovery and social inclusion for occupational therapy practice in the UK. Ment Health Soc Incl.

[48] Peters BC, Wood W, Hepburn S, Bundy A (2020). Pilot study: occupational therapy in an equine environment for youth with autism. OTJR Occup Participation Health.

[49] Kearns Murphy C, Shiel A (2021). Evaluation of an intensive occupational therapy intervention to facilitate independent living and improve occupational performance and participation. Results of a longitudinal case study design. Occup Ther Mental Health.

[50] Verikios D, Hitch D, Andriske L (2016). Achieving occupational goals with the TAPit: a case study. Int J Ther Rehabil.

[51] Dibsdall L. A realist synthesis and evaluation of the role and impact of occupational therapists in reablement services. University of the West of England. 2019.

[52] Hurst HS (2020). Using the canadian model of occupational performance in occupational therapy practice: a case study enquiry.

[53] Hamel J, Dufour S, Fortin D (1993). Case study methods.

[54] Thorne S (2012). What’s in a case?. Nurs Inq.

[55] Blatter J, Haverland M (2012). Designing case studies: explanatory approaches in small-N research.

[56] Green M, Barstow B, Vogtle L (2018). Lighting as a compensatory strategy for acquired visual deficits after stroke: two case reports. Am J Occup Ther.

[57] Proffitt RM, Henderson W, Scholl S, Nettleton M, Silverman Lee (2018). voice treatment BIG Â® for a person with strok. Am J Occup Ther.

[58] Longpre SM, Polo KM, Baxter MF (2020). A personal perspective on daily occupations to counteract cancer related fatigue: a case study. Open J Occup Ther.

[59] Hyett N, Kenny A, Dickson-Swift V (2019). Re-imagining occupational therapy clients as communities: presenting the community-centred practice framework. Scand J Occup Ther.

[60] Metcalfe V, Egan M, Sauvé-Schenk K (2019). LSVT BIG in late stroke rehabilitation: a single-case experimental design study. Can J Occup Ther.

[61] Lawson S, Tang Z, Feng J (2017). Supporting Stroke motor recovery through a mobile application: a pilot study. Am J Occup Ther.

[62] Belliveau D, Belliveau I (2016). Use of Occupational Performance Coaching for stroke survivors (OPC-Stroke) in late rehabilitation: a descriptive case study. Open J Occup Ther (OJOT).

[63] Belliveau D, Belliveau I, Camire-Raymond A, Kessler D, Egan M (2016). Use of Occupational Performance Coaching for stroke survivors (OPC-Stroke) in late rehabilitation: a descriptive case study. Open J Occup Ther.

[64] Carlstedt E, Iwarsson S, Stahl A, Pessah-Rasmussen H, Lexell EM (2017). BUS TRIPS-A self-management program for people with cognitive impairments after stroke. Int J Environ Res Public Health.

[65] Park HI, Park HY, Yoo E, Han A (2020). Impact of family-centered early intervention in infants with autism spectrum disorder: a single-subject design. Occup Ther Int.

[66] Babik I, Cunha AB, Lobo MA (2021). Assistive and rehabilitative effects of the Playskin Lift TM exoskeletal garment on reaching and object exploration in children with arthrogryposis. Am J Occup Ther.

[67] Henning B, Cordier R, Wilkes-Gillan S, Falkmer T (2016). A pilot play-based intervention to improve the social play interactions of children with autism spectrum disorder and their typically developing playmates. Aust Occup Ther J.

[68] Kim MK, Kim DJ (2018). Effects of oral stimulation intervention in newborn babies with Cri du Chat syndrome: single-subject research design. Occup Ther Int.

[69] Gustafsson L, Patterson E, Marshall K, Bennett S, Bower K (2016). Efficacy of compression gloves in maintaining edema reductions after application of compression bandaging to the stroke-affected upper limb. Am J Occup Ther.

[70] Gimeno H, Polatajko HJ, Lin JP, Cornelius V, Brown RG (2021). Cognitive strategy training in childhood-onset movement disorders: replication across therapists. Front Pediatr.

[71] Lorenzo T, McKinney V, Bam A, Sigenu V, Sompeta S (2019). Mapping participation of disabled youth in sport and other free-time activities to facilitate their livelihoods development. Br J Occup Ther.

[72] Fischl C, Blusi M, Lindgren H, Nilsson I (2020). Tailoring to support digital technology-mediated occupational engagement for older adults–a multiple case study. Scand J Occup Ther.

[73] Johanson S, Markström U, Bejerholm U (2019). Enabling the return-to-work process among people with affective disorders: a multiple-case study. Scand J Occup Ther.

[74] Sonday A, Gretschel P (2016). Empowered to play: a case study describing the impact of powered mobility on the exploratory play of disabled children. Occup Ther Int.

[75] Kassberg AC, Prellwitz M, Malinowsky C, Larsson-Lund M (2016). Interventions aimed at improving the ability to use everyday technology in work after brain injury. Scand J Occup Ther.

[76] Umeda CJ (2017). Community cultural arts participation through sensory friendly theatre: parent and organization experiences and perspectives.

[77] Peruzzolo DL, Barbosa DM, de Souza APR (2018). Occupational Therapy and babies treatment in premature intervention from a hypothesis of psychomotor functioning: single case study. Bra J Occup Ther.

[78] Haines D, Wright J, Comerasamy H (2018). Occupational therapy empowering support workers to change how they support people with profound intellectual and multiple disabilities to engage in activity. J Pol Pract Intellect Disabil.

[79] Pretorius M (2018). The contribution of occupational therapy in the holistic management of a child with tetra-amelia syndrome.

[80] Suarez MA, Bush E (2020). Pilot study of the just right challenge feeding protocol for treatment of food selectivity in children. Open J Occup Ther.

[81] CândidoSoares JC, de Moraes BLC, dos Santos Couto Paz CC, de Castro Magalhães L (2019). Influence of the microsoft kinect® games on the motor and functional performance of a child with developmental coordination disorder. Bra J Occup Ther.

[82] DiasdaSilvia T, da Conceição KF, de Oliveira AIA, da Silva RLM (2017). The contributions of game therapy concerning motor performance of individual with cerebral palsy. Bra J Occup Ther Cadernos Brasileiros de Terapia Ocupacional.

[83] Joyce J, Warren A (2016). A case study exploring the influence of a gardening therapy group on well-being. Occup Ther Mental Health.

[84] Kent C, Cordier R, Joosten A, Wilkes-Gillan S, Bundy A (2020). Can I join in? Multiple case study investigation of play performance generalisation for children with autism spectrum disorder from dyad to triad. Aust Occup Ther J.

[85] Khoshbakht M, Raji P, Ansari NN, Mahmodian M (2021). Impact of somatosensory interventions on upper limb function in children with hemiplegic cerebral palsy: a single-subject design study. Int J Ther Rehabil.

[86] Mohammadi A, Mehraban A, Damavandi S (2017). Effect of play-based occupational therapy on symptoms of hospitalized children with cancer: a single-subject study. Asia Pac J Oncol Nurs.

[87] Alterio CJ (2019). Clinically orientated theory for occupational therapy.

[88] Giencke Kimball J, Cao L, Draleau KS (2018). Efficacy of the Wilbarger therapressure program (TM) to modulate arousal in women with post-traumatic stress disorder: a pilot study using salivary, cortisol and behavioral measures. Occup Ther Ment Health.

[89] Go EJ, Lee SH (2016). Effect of sensorimotor stimulation on chronic stroke patients’ upper extremity function: a preliminary study. J Phys Ther Sci.

[90] Hejazi-Shirmard M, Taghizadeh G, Azad A, Lajevardi L, Rassafiani M (2020). Sensory retraining improves light touch threshold of the paretic hand in chronic stroke survivors: a single-subject A-B design. Somatosens Mot Res.

[91] Cruz G, Petrie S, Goudie N, Kersel D, Evans J (2016). Text messages reduce memory failures in adults with brain injury: a single-case experimental design. Br J Occup Ther.

[92] Gervais MÈ, Couture M, le Blanc S, Blanchet S, Gagné MÈ, Ouellet MC (2017). Evaluation of cognitive functioning in the context of rehabilitation for visual impairment in older adults: a case series. Phys Occup Ther Geriatr.

[93] Teixeira GRA, de Alves ACJ (2021). Occupational therapy intervention in paralympic sport: a look at low-cost assistive technology for wheelchair rugby. Disabil Rehabil: Assist Technol.

[94] Carey A, Cockburn L, Langlois S (2019). Investigating the provision of occupational therapy services: a case study. Can J Occup Ther.

[95] Golisz K, Waldman-Levi A, Swierat RP, Toglia J (2018). Adults with intellectual disabilities: case studies using everyday technology to support daily living skills. Br J Occup Ther.

[96] Sørlie C, Cowan M, Chacksfield J, Vaughan E, Atler KE (2020). Occupation-focused assessment in eating disorders: preliminary utility. Occup Ther Ment Health.

[97] Ribeiro J, Mira E, Lourenço I, Santos M, Braúna M (2019). The intervention of occupational therapy in drug addiction: a case study in the comunidade terapêutica clínica do outeiro – Portugal. Ciencia e Saude Coletiva.

[98] Hyett N, Kenny A, Dickson-Swift V (2017). Approaches for building community participation: a qualitative case study of Canadian food security programs. OTJR.

[99] Peny-Dahlstrand M, Bergqvist L, Hofgren C, Himmelmann K, Öhrvall AM (2020). Potential benefits of the cognitive orientation to daily occupational performance approach in young adults with spina bifida or cerebral palsy: a feasibility study. Disabil Rehabil.

[100] Carey H (2021). An integrative review of case study methodologies in occupational therapy publications. Bra J Occup Ther.

[101] Lane JD, Ledford JR, Gast DL (2017). Single-case experimental design: current standards and applications in occupational therapy. Am J Occup Ther.

[102] Alpi KM, Evans JJ (2019). Distinguishing case study as a research method from case reports as a publication type. J Med Libr Assoc.

[103] Pierce D (2012). Promise. J Occup Sci.

[104] Kuhn TS, Kruger L, Dalston LJ, Heidelberger M (1987). What are scientific revolutions?. The probabilistic revolution, Ideas in history.

[105] Hui C, Snider L, Couture M (2016). Self-regulation workshop and occupational performance coaching with teachers: a pilot study. Can J Occup Ther.

[106] Gustafsson L, Lunnon J, Hoyle M, Marshall K, Bower K (2016). Single-case-design study of finger-to-axilla compression bandaging for edema of the hemiplegic upper limb. Am J Occup Ther.

[107] Suder R, Fu MJ, Knutson J, Curby A (2016). Contralaterally controlled functional electrical stimulation and hand therapy video games for cerebral palsy. Am J Occup Ther.

[108] Yoshihiro N, Ito E (2017). Effect of passive limb activation by Functional Electrical Stimulation on wheelchair driving in patients with unilateral spatial neglect: a case study. Hong Kong J Occup Ther.

[109] Association AOT (2021). Standards of practice for occupational therapy. Am J Occup Ther.

[110] Occupational Therapy Board of Australia. Australian occupational therapy competency standards 2018. 2018. https://www.occupationaltherapyboard.gov.au/codes-guidelines/competencies.aspx Accessed 12 Feb 2022.

[111] Cavalcanti A, Amaral MF, Silva e Dutra FCM, Santos AVF, Licursi LA, Silveira ZC (2020). Adaptive eating device: performance and satisfaction of a person with Parkinson’s disease. Can J Occup Ther.

[112] Gontijo DT, de Sena e Vasconcelos AC, Monteiro RJS, Facundes VLD, de FátimaCordeiroTrajano M, de Lima LS (2016). Occupational therapy and sexual and reproductive health promotion in adolescence: a case study. Occup Ther Int.

[113] Brett S, Hitch D, Woodhead G, Simmons M (2016). Evaluation of a sensory modulation group in a community youth mental health setting. Early Interv Psychiatry.

[114] Do JH, Yoo EY, Jung MY, Park HY (2016). The effects of virtual reality-based bilateral arm training on hemiplegic children’s upper limb motor skills. NeuroRehabilitation.

[115] Fleming MH (1991). The therapist with the three-track mind. Am J Occup Ther.

[116] Dougherty DA, Toth-Cohen SE, Tomlin GS (2016). Beyond research literature: occupational therapists’ perspectives on and uses of “evidence” in everyday practice. Can J Occup Ther.

[117] Turpin M, Iwama M (2011). Using occupational therapy models in practice: a field guide.

[118] Arntzen C (2018). An embodied and intersubjective practice of occupational therapy. OTJR Occup Participation Health.

[119] Fleming MH (1991). Clinical reasoning in medicine compared with clinical reasoning in occupational therapy. Am J Occup Ther.

[120] SAGE Publications. Clinical Case Studies. 2022. Available from: https://journals.sagepub.com/home/ccs Accessed 7 Apr 2022.

[121] Japanese Association of Occupational Therapists. JAOT Activities. 2020. Available from: https://www.jaot.or.jp/en/activities/ Accessed 7 Apr 2022.

[122] Kiresuk T, Sherman R (1968). Goal attainment scaling: general method for evaluating comprehensive community mental health programs. Community Ment Health J.

[123] Kearns Murphy C, Shiel A (2019). Developing clear and useful scoring procedures for goal attainment scaling in longitudinal case studies. Occup Ther Ment Health.

[124] Kearns Murphy C, Shiel A (2019). Institutional injustices? Exploring engagement in occupations in a residential mental health facility. J Occup Sci.

