# Supplementary material for: Exploring the contribution of case study research to the evidence base for occupational therapy: a scoping review
Source: Syst Rev. 2023 Jul 31;12:132. doi: 10.1186/s13643-023-02292-4 (PMC10388505; doi:10.1186/s13643-023-02292-4)
Supplement: Supplementary file 2 — Additional file 2. Search strategy. [file 13643_2023_2292_MOESM2_ESM.docx]

**AMED (Ebsco)**

Search conducted June 2020. Default field entry: abstract, title and keywords

| **Search** | **Query** | **Records retrieved** |
| --- | --- | --- |
| 1 | (SU “occupational therapy”) OR (SU “occupational therapists”) | 11, 361 |
| 2 | "occupational therap*" | 15, 717 |
| 3 | “occupational science” | 395 |
| 4 | SO (“Occupational science”) OR (SO “occupational therapy”) | 11, 654 |
| 5 | S1 OR S2 OR S3 OR S4 | 15, 871 |
| 6 | (SU “case report”) | 8, 204 |
| 7 | “case study research” | 26 |
| 8 | “single-case” | 555 |
| 9 | “single case” | 555 |
| 10 | “Single-case experimental design” | 34 |
| 11 | SCED | 3 |
| 12 | “multiple-case” | 82 |
| 13 | “multiple case” | 82 |
| 14 | “case design*” | 81 |
| 15 | “case-report*” | 11, 279 |
| 16 | “case report*” | 11, 279 |
| 17 | “case stud*” | 3, 805 |
| 18 | “case-stud*” | 3, 805 |
| 19 | “single-subject” | 363 |
| 20 | “single subject” | 363 |
| 21 | “N-of-1” | 25 |
| 22 | “Qualitative case study method*” | 9 |
| 23 | QCSM | 0 |
| 24 | S6 OR S7 OR S8 OR S9 OR S10 OR S11 OR S12 OR S13 OR S14 OR S15 OR S16 OR S17 Or S18 OR S19 OR S20 ORS21 OR S22 | 14, 614 |
| 25 | S24 AND S5 | 791 |
|  | S25 with limiters applied: English language, published after 1990. | 684 |
|  | Updated July 2021 = 7 results | 691 |

**CINAHL (Ebsco)**

Search conducted June 2020. Default field entry: abstract, title and keywords

| **Search** | **Query** | **Records retrieved** |
| --- | --- | --- |
| 1 | (MH "Occupational Therapy") OR (MH "Occupational Therapists") | 31, 100 |
| 2 | "occupational therap*" | 49, 941 |
| 3 | “occupational science” | 818 |
| 4 | (SO “occupational science”) OR (SO “occupational therapy”) | 43, 042 |
| 5 | S1 OR S2 OR S3 OR S4 | 64, 699 |
| 6 | (MH "Case Studies") | 27, 375 |
| 7 | “case study research” | 421 |
| 8 | “single-case” | 3, 861 |
| 9 | “single case” | 3, 861 |
| 10 | “Single-case experimental design” | 209 |
| 11 | SCED | 48 |
| 12 | “multiple-case” | 1, 358 |
| 13 | “multiple case” | 1, 358 |
| 14 | “case design*” | 489 |
| 15 | “case-report*” | 80, 348 |
| 16 | “case report*” | 80, 348 |
| 17 | “case stud*” | 83, 106 |
| 18 | “case-stud*” | 83, 106 |
| 19 | “single-subject” | 1, 811 |
| 20 | “single subject” | 1, 811 |
| 21 | “N-of-1” | 230 |
| 22 | “Qualitative case study method*” | 105 |
| 23 | QCSM | 1 |
| 24 | S6 OR S7 OR S8 OR S9 OR S10 OR S11 OR S12 OR S13 OR S14 OR S15 OR S16 OR S17 Or S18 OR S19 OR S20 ORS21 OR S22 OR S23 | 163, 952 |
| 25 | S24 AND S5 | 2, 096 |
|  | S25 with limiters applied: English language, published after 1990. | 1, 669 |
|  | Updated July 2021 = 111 results | 1, 780 |

**Medline (Ebsco)**

Search conducted June 2020. Default field entry: abstract, title and keywords

| **Search** | **Query** | **Records retrieved** |
| --- | --- | --- |
| 1 | (MH “Occupational Therapists”) OR (MH “Occupational Therapy”) | 13, 311 |
| 2 | “occupational therap*” | 31, 996 |
| 3 | “occupational science” | 1, 619 |
| 4 | (SO “Occupational science”) OR (SO “occupational therapy”) | 11, 578 |
| 5 | S1 OR S2 OR S3 OR S4 | 34, 514 |
| 6 | (MH “case reports”) | 0 |
| 7 | “case study research” | 311 |
| 8 | “single-case” | 9, 404 |
| 9 | “single case” | 9, 404 |
| 10 | “Single case experimental design” | 236 |
| 11 | SCED | 108 |
| 12 | “multiple-case” | 1, 739 |
| 13 | “multiple case” | 1, 739 |
| 14 | “case design*” | 576 |
| 15 | “case-report*” | 374, 677 |
| 16 | “case report*” | 374, 677 |
| 17 | “case stud*” | 104, 405 |
| 18 | “case-stud*” | 104, 405 |
| 19 | “single-subject” | 3, 171 |
| 20 | “single subject” | 3, 171 |
| 21 | “N-of-1” | 432 |
| 22 | “Qualitative case study method*” | 103 |
| 23 | QCSM | 1 |
| 24 | S6 OR S7 OR S8 OR S9 OR S10 OR S11 OR S12 OR S13 OR S14 OR S15 OR S16 OR S17 Or S18 OR S19 OR S20 ORS21 OR S22 OR S23 | 483, 433 |
| 24 | S24 AND S5 | 1, 128 |
| 25 | S24 with limiters applied: English language, published after 1990. | 1, 016 |
|  | Updated July 2021 = 177 results | 1,193 |

**EMBASE (OVID)**

Search conducted June 2020.

| **Search** | **Query** | **Records retrieved** |
| --- | --- | --- |
| 1 | exp occupational therapy/ | 21, 526 |
| S2 | exp occupational therapist/ | 6, 655 |
| S3 | occupational therap$.ti,ab,kw. | 20, 698 |
| S4 | occupational science.ti,ab,kw | 174 |
| S5 | (occupational therapy in health care or occupational therapy in mental health or occupational therapy international or "occupational therapy journal of research").jn. | 2273 |
| S6 | S1 OR S2 OR S3 OR S4 OR S5 | 31, 440 |
| S7 | exp case report/ | 248, 3667 |
| S8 | exp case study/ | 69, 785 |
| S9 | case study research.ti,ab,kw. | 371 |
| S10 | single-case.ti,ab,kw. | 13, 947 |
| S11 | single case.ti,ab,kw. | 13, 947 |
| S12 | Single-case experimental design.ti,ab,kw | 336 |
| S13 | SCED.ti,ab,kw. | 119 |
| S14 | multiple-case.ti,ab,kw. | 2205 |
| S15 | multiple case.ti,ab,kw. | 2205 |
| S16 | case design$.ti,ab,kw. | 824 |
| S17 | case-report$.ti,ab,kw. | 493, 169 |
| S19 | case report$.ti,ab,kw. | 493, 169 |
| S20 | case stud$.ti,ab,kw. | 124, 812 |
| S21 | case-stud$.ti,ab,kw. | 124, 812 |
| S22 | single-subject.ti,ab,kw. | 4310 |
| S23 | single subject.ti,ab,kw. | 4310 |
| S24 | “n-o#-1”.ti,ab,kw. | 811 |
| S25 | qualitative case study method$.ti,ab,kw. | 124 |
| S26 | QCSM.ti,ab,kw. | 1 |
| S27 | 8 or 9 or 10 or 11 or 12 or 13 or 14 or 15 or 16 or 17 or 18 or 19 or 20 or 21 or 22 or 23 or 24 or 25 or 26 | 269, 6335 |
| S28 | 7 and 27 | 3279 |
| S29 | S28 with limiters applied: English language, published after 1990. | 2955 |
|  | Updated July 2021 = 10 results | 2965 |

**PsychInfo (ProQuest)**

Search conducted June 2020.

| **Search** | **Query** | **Records retrieved** |
| --- | --- | --- |
| 1 | mainsubject ("occupational therapy" OR "occupational therapists") | 8837 |
| 2 | NOFT “occupational therap*" | 21, 023 |
| 3 | “occupational science” | 1, 679 |
| 4 | PUB (“occupational science” OR “occupational therapy”) | 9, 781 |
| 5 | S1 OR S2 OR S3 OR S4 | 21, 115 |
| 6 | mainsubject ("case report") | 22, 945 |
| 7 | NOFT “case study research” | 1, 333 |
| 8 | NOFT “single-case” | 7, 654 |
| 9 | NOFT “single case” | 7, 654 |
| 10 | NOFT “Single-case experimental design” | 392 |
| 11 | NOFT “SCED” | 68 |
| 12 | NOFT “multiple-case” | 4, 106 |
| 13 | NOFT “multiple case” | 4, 106 |
| 14 | NOFT “case design*” | 1, 236 |
| 15 | NOFT “case-report*” | 46, 431 |
| 16 | NOFT “case report*” | 46, 431 |
| 17 | NOFT “case stud*” | 176, 840 |
| 18 | NOFT “case-stud*” | 177, 388 |
| 19 | NOFT “single-subject” | 3, 274 |
| 20 | NOFT “single subject” | 3, 274 |
| 21 | NOFT “N-of-1” | 181 |
| 22 | NOFT “Qualitative case study method*” | 85 |
| 23 | NOFT “QCSM” | 1 |
| 24 | S6 OR S7 OR S8 OR S9 OR S10 OR S11 OR S12 OR S13 OR S14 OR S15 OR S16 OR S17 Or S18 OR S19 OR S20 ORS21 OR S22 OR S23 | 191, 550 |
| 25 | S24 AND S5 | 1, 193 |
|  | S25 with limiters applied: English language, published after 1990. | 1, 024 |
|  | Updated July 2021 = 14 results | 1, 038 |

**Web of Science**

Search conducted June 2020.

| **Search** | **Query** | **Records retrieved** |
| --- | --- | --- |
| S1 | TS="occupational therap*" | 16,037 |
| S2 | TS=“occupational science” | 405 |
| S3 | SO=“occupational science” OR SO=“occupational therapy” | 0 |
| S4 | S1 OR S2 OR S3 | 16, 271 |
| S5 | TS=“case study research” | 2, 957 |
| S6 | TS= “single-case” | 12, 266 |
| S7 | TS= “single case” | 12, 266 |
| S8 | TS=“Single-case experimental design” | 316 |
| S9 | TS=SCED | 337 |
| S10 | TS= “multiple-case” | 6, 036 |
| S11 | TS= “multiple case” | 6,036 |
| S12 | TS= “case design*” | 1, 981 |
| S13 | TS= “case-report*” | 298, 027 |
| S14 | TS= “case report*” | 298, 027 |
| S15 | TS= “case stud*” | 541, 420 |
| S16 | TS= “case-stud*” | 541, 420 |
| S17 | TS= “single-subject” | 4, 709 |
| S18 | TS= “single subject” | 4, 709 |
| S19 | TS= “N-of-1” | 1, 120 |
| S20 | TS= “Qualitative case study method*” | 319 |
| S21 | TS=QCSM | 4 |
| S22 | S5 OR S6 OR S7 OR S8 OR S9 OR S10 OR S11 OR S12 OR S13 OR S14 OR S15 OR S16 OR S17 Or S18 OR S19 OR S20 OR S21 | 850, 188 |
| S23 | S22 AND S4 | 600 |
| S24 | S23 with limiters applied: English language, published after 1990. | 560 |
|  | Updated July 2021 – 60 results | 620 |

**Open Access Theses and Dissertations (OATD)**

Search conducted June 2020. Abstract field enabled

| **Search Terms** | **Results** |
| --- | --- |
| occupational therapy AND case study | 51 |
| Updated July 2021 = 3 results | 54 |

**OpenGrey**

Search conducted June 2020.

| **Search Terms** | **Results** |
| --- | --- |
| ("Occupational therapy" OR "occupational therapist" OR "occupational therapists" OR "occupational science") AND ("case study research" OR "single-case" OR "single case" OR "single case experimental design" OR "SCED" OR "multiple case" OR "multiple-case" OR "case design" OR "case designs" OR "case-report" OR "case-reports" OR "case report" OR "case reports" OR "case study" OR "case studies" OR "case-stud" OR "case-studies" OR "single-subject" OR "single subject" OR "N-of-1" OR "Qualitative case study method" OR "qualitative case study methods" OR QCSM) lang:"en" | 7 |
| Updated July 2021 = 0 results | 7 |

**Occupational therapy publications**

Search conducted June 2020.

| **Magazine** | **Date range searched** | **Results** |
| --- | --- | --- |
| Occupational Therapy News (UK) | Aug 2016–June 2020 | 0 |
| Occupational Therapy Practice (USA) | Jan 2016–Oct 2018 | 0 |
| Occupational Therapy Now (Canada) | Jan 2016–April 2020 | 0 |
|  | Updated July 2021 = 0 results | 0 |

**Ethos**

Search conducted June 2020. Abstract field enabled.

| **Search Terms** | **Results** |
| --- | --- |
| ("occupational therapy" OR "occupational therapist" OR "occupational science" OR "occupational therapists") AND ("case study" OR "case studies" OR "single subject" OR "case design" OR "case report" OR "case reports" OR "multiple case" OR "single case") | 3 |
| Updated July 2021 = 20 results | 23 |

**OTDBase**

Search conducted June 2020.

| **Search terms and fields** | **Results** |
| --- | --- |
| Title field - Case study  Abstract - single subject | 2 |
| Title field - Case studies  Abstract - single subject | 0 |
| Title field – Case-study  Abstract - single subject | 2 |
| Title field - Case study  Abstract – single-subject | 2 |
| Title field - Case studies  Abstract – single-subject | 2 |
| Title field – Case-study  Abstract – single-subject | 2 |
| Title field – Case study  Abstract – single case | 8 |
| Title field – Case studies  Abstract – single case | 1 |
| Title field – Case-study  Abstract – single case | 8 |
| Title field – Case study  Abstract – single-case | 8 |
| Title field – Case studies  Abstract – single-case | 1 |
| Title field – Case-study  Abstract – single-case | 8 |
| Total included after duplicates removed | 11 |

Not updated in 2021—library services not available by RCOT due to covid19 pressures

**Google**

Search conducted June 2020.

| **Search Terms** | **Results** |
| --- | --- |
| "occupational therapy" OR "occupational science" AND "case study" OR "single case" | As per protocol, first 50 results screened for eligibility. |
| Updated July 2021 = 0 new relevant results |  |

**Google Scholar:**

Search conducted June 2020. All in title, custom range 1990-current

| **Search Terms** | **Results** |
| --- | --- |
| "occupational therapy" OR "occupational science" AND "case study" OR "single case" | As per protocol, first 50 results screened for eligibility. |
| Updated July 2021 = 1 new result |  |
