# Supplementary material for: Exploring the contribution of case study research to the evidence base for occupational therapy: a scoping review
Source: Syst Rev. 2023 Jul 31;12:132. doi: 10.1186/s13643-023-02292-4 (PMC10388505; doi:10.1186/s13643-023-02292-4)
Supplement: Supplementary file 3 — Additional file 3. Data extraction instrument (Empirical studies). [file 13643_2023_2292_MOESM3_ESM.docx]

| Citation details | | | | | | Case study definition | | |  |
| --- | --- | --- | --- | --- | --- | --- | --- | --- | --- |
| Study | Author | Year | Country | Information source | Journal title | Case study definition | Justification for case study | Ethics approval | Study aim |
| 1 |  |  |  |  |  |  |  |  |  |
| 2 |  |  |  |  |  |  |  |  |  |

|  | Context | | | | | | | Methodology characteristics | | | | | |
| --- | --- | --- | --- | --- | --- | --- | --- | --- | --- | --- | --- | --- | --- |
| Study | Population age | Population Diagnosis | Context of research | Intervention | Outcome measure (s) | Findings | Implications for practice | Case (s)  N = | Case(s) descript-ion | Data collection | Data analysis | Case study design |  |
| 1 |  |  |  |  |  |  |  |  |  |  |  |  |  |
| 2 |  |  |  |  |  |  |  |  |  |  |  |  |  |
