# Supplementary material for: Exploring the contribution of case study research to the evidence base for occupational therapy: a scoping review
Source: Syst Rev. 2023 Jul 31;12:132. doi: 10.1186/s13643-023-02292-4 (PMC10388505; doi:10.1186/s13643-023-02292-4)
Supplement: Supplementary file 4 — Additional file 4. Data extraction instrument (Non-empirical studies). [file 13643_2023_2292_MOESM4_ESM.docx]

| Citation details | | | | | | Case study definition | | |  |
| --- | --- | --- | --- | --- | --- | --- | --- | --- | --- |
| Study | Author | Year | Country | Information source | Journal title | Study aim | Case study definition | Explanation of methodology | Explanation of ‘case’ |
| 1 |  |  |  |  |  |  |  |  |  |
| 2 |  |  |  |  |  |  |  |  |  |

|  | Methodology characteristics | | | | | |
| --- | --- | --- | --- | --- | --- | --- |
| Study | Reported strengths | Reported limitation | Explanation of data collection | Explanation of data analysis | Key findings | Implications for Practice |
| 1 |  |  |  |  |  |  |
| 2 |  |  |  |  |  |  |
