# Supplementary material for: Exploring the contribution of case study research to the evidence base for occupational therapy: a scoping review
Source: Syst Rev. 2023 Jul 31;12:132. doi: 10.1186/s13643-023-02292-4 (PMC10388505; doi:10.1186/s13643-023-02292-4)
Supplement: Supplementary file 5 — Additional file 5. Characteristics of included empirical studies. [file 13643_2023_2292_MOESM5_ESM.docx]

| Study (Country) | Study Aim | Case study design | (N) Case description / Context | Data collection / Data analysis | Findings / Implications for Practice |
| --- | --- | --- | --- | --- | --- |
| Babik et al, 2021 (USA) | Evaluate longitudinal assistive and rehabilitative effects of the Playskin Lift™ on reaching and object exploration abilities in children with arthrogryposis | ABA single-case longitudinal design | (16) children with arthrogryposis / Community | Quantitative / Statistical analysis | Improved performance when wearing the Playskin within sessions and improved independent performance after the intervention |
| Belliveau et al, 2016 (Canada) | Explore efficacy of occupational performance coaching for stroke survivors on participation levels of adults in the later stage of stroke rehabilitation | Descriptive case study | (1) One participant in later stages of stroke rehabilitation / Not reported | Mixed Methods / Conventional qualitative content analysis. | OPC-Stroke was valued by the participant and shows promise for improving goal performance and satisfaction. |
| Bulkeley, 2016 (Australia) | Explore effectiveness of sensory-based family-centred coaching approach to changing problematic routines for young children with autism. | Single-case experimental design (SCED) | (3) Three mothers and young children with autism / community | Quantitative / Visual analysis and descriptive analysis | The sensory-based, family-centred coaching approach showed promise for changing sensory-related problem behaviours of young children with autism. |
| Brett et al 2016 (Australia) | Evaluate implementation of a sensory modulation group in a specialized youth mental health service | Mixed methods case study design | (Not reported) Youth in a specialized mental health service / Not reported | Mixed methods / Not reported | Not reported |
| Cândido Soares et al, 2019 (Brazil) | Evaluate influence of the use of Microsoft Kinect^®^ games on motor and functional performance of a child with DCD | A1-B-A2 study | (1) Individual male / Not reported | Quantitative / Four stage evaluations | Microsoft Kinect^®^ proved to be a viable alternative to stimulate motor performance, besides being motivating for the participant |
| Capistran & Martini, 2016 (Canada) | Determine whether CO-OP improves occupational performance | Single-subject multiple baseline | (4) Children with DCD / Not reported | Quantitative / Visual inspection of graphs, statistical analyses descriptive analysis | CO-OP is a promising intervention for obtaining inter-task transfer in children, but that in-therapy sessions may not be sufficient |
| Carey et al, 2019 (Canada) | Uncover what is known about the case of occupational therapy in the context of mental health services in Saskatchewan | Instrumental case study | (1) Occupational therapy in Saskatchewan / NA | Qualitative / Thematic analysis | illuminates how restricted access to, limited awareness of, and ineffective advocacy for occupational therapy impacts mental health services in Saskatchewan |
| Carlstedt et al, 2017 (Sweden) | Investigate the feasibility of a new self-management intervention (BUS TRIPS) and potential contributions to an improved ability to travel by bus for people with cognitive impairments after stroke | Multiple case study | (5) Participants (two women, three men) / Not reported | Mixed methods / Cross case analysis; content analysis and within case analysis | BUS TRIPS intervention is feasible and can potentially contribute to an improved ability to travel by bus for the target group. |
| Carroll, 2020 (USA) | Examined the use of a family occupation-centred coaching intervention to support two parents’ implementation of evidence-based social interaction strategies in their home with their toddler with autism spectrum disorder. | Two-single-case changing criterion designs | (3) Caucasian family / Community | Quantitative / Statistical analysis | Intervention yielded a 55.26% improvement in the quality of social interactions for the family, 69.27% for the mother, 64.07% for the father, and 30.69% for the child. |
| Cavalcanti et al, 2016 (Brazil) | Investigate if a locally made eating adaptive device helps a person with PD to improve their feeding performance | Single-case ABC-type experimental design | (1) 60-year-old man with PD / Community | Quantitative / Statistical analysis and visual analysis. | Performance and satisfaction increased significantly after the introduction of the adaptive eating device. |
| Cruz et al, 2016 (UK) | Evaluate the uptake of a simple, inexpensive memory aid – SMS notifications sent through Google Calendar – to reduce everyday memory failures in two patients and to identify which factors contributed to the outcome | Single-case experimental design multiple baseline | (2) participants attending a community brain injury rehab service / Community | Quantitative / Pre and post results comparison | Improved self-perception of performance and satisfaction levels. Technology that is ubiquitous to daily life can be effectively integrated into cognitive rehabilitation for people with traumatic brain injury to increase participation |
| da Silva Dias et al, 2017 (Brazil) | Analyse the use of game therapy as a motor (re) habilitation method for individuals with CP | Exploratory and quantitative field in which case study was used | (1)12-year-old boy with CP / Community | Quantitative / Comparing scores pre and post intervention | Game therapy, specifically through the Aladdin Magic Racer game (the Nintendo Wii), can be suggested as a motor (re) habilitation method for CP subjects. |
| Dibsdall, 2019 (UK) | Advance understanding of the role of occupational therapists in reablement services including the factors that influence their practice | Multiple case study | (3) Reablement services with occupational therapy working in them / Community | Qualitative / Explanation building (pattern matching), thematic analysis & member checking | It is recommended that organizations employ occupational therapists in reablement. |
| Do et al, 2016 (Korea) | Verify whether virtual reality-based bilateral upper limb exercise is effective for improvement of hemiplegic cerebral palsy children’s affected upper limb functions and bilateral hand coordination ability | Single-subject experimental design | (3) Children with hemiplegic CP / Outpatient clinic | Quantitative / Visual graphs | After virtual reality-based bilateral arm training, improvement occurred in upper limb motor skills on the affected sides, and in bilateral coordination ability, for all of the research subjects. |
| Fields, 2017 (USA) | Investigate the influence an equine-assisted intervention, Riding in the Moment (RM), on quality of life (QoL) for institutionalized adults with dementia | Exploratory mixed methods case study | (6) People with dementias’ experiences in activity situations / Community | Mixed method. / Statistical testing, descriptive statistics, qualitative description, convergence of data | RM may be an effective environmental intervention that allows institutionalized adults with dementia a means to return to nature. |
| Fischl et al, 2020 (Sweden) | Explore how tailoring to support older adults’ engagement in DT-mediated occupations could be schematized | Multiple case study methodology | (9) Three male and six female participants, / Community | Mixed methods / Within case, cross-case synthesis | The proposed scheme for tailoring could contribute to occupational therapists’ knowledge on how to support older adults’ engagement in contemporary occupations. |
| Gartz et al, 2021 (USA)  *(linked report; Gartz, 2018)* | Compare the effectiveness of component based, occupation-based, and a combined intervention for visual- scanning to improve occupational performance | Exploratory single-case study design | (1) 55-year-old female 7 years post stroke / Community | Quantitative / Comparison of scores, visual analysis | A programme specifically targeting visual scanning compensation to improve occupational performance for individuals poststroke should include both component-based treatment to compensate for continuing deficits and occupation-based treatment to promote generalization and transfer of component-based skills |
| Gee et al, 2016 (USA) | Explore the efficacy of weighted blankets with children with an autism spectrum disorder and sleep disturbances | ABA single subject design | (2) Children with an autism spectrum disorder and sensory over-responsivity / Community | Quantitative / Visual analysis | Overall findings demonstrated minimal improvement of the measured constructs related to sleep quality in the two participants. |
| George-Paschal & Bowen, 2019 (USA) | Evaluate the benefits of an OA-based mentoring programme for court-appointed adolescents in a juvenile drug court | Descriptive case study methodology | (6) Youth enrolled in a juvenile drug court programme/ Community | Mixed methods / Descriptive analysis | OA-based mentoring programme implemented in this study was found to be beneficial for adolescent participants assigned to a juvenile drug court. Five of the six participants showed an increase in relative mastery with regard to their self-identified goal(s). |
| Gervais et al, 2017 (Canada) | Verify the consistency between the Montreal Cognitive Assessment (MoCA) and the Mini-Mental State Examination (MMSE) and the therapist’s observations of elderly individuals in low-vision rehabilitation | Single-case research design also says case series | (6) Elderly individuals who receiving occupational therapy / Community | Mixed methods / Visual analysis | Standardized cognitive tests had limited utility to predict the complexity of LVR. Cognitive difficulties do not preclude rehabilitation for even severe visual impairment in elderly persons and does not imply significantly longer or more intense rehabilitation. |
| Ghorbani et al, 2017 (Iran) | Determine whether the CO-OP approach improves motor skills and achievement in motor-based occupational performance goals in children with CP | Multiple baseline single-case experimental design | (5) Children with CP / Community | Quantitative / Visual analysis, statistical analysis | CO-OP intervention can be helpful in improving motor skills and achieving self-identified, motor-based goals in children with CP |
| Giencke Kimball et al, 2018 (USA) | Assesses the efficacy of using the Wilbarger Therapressure Program™ to modulate the arousal and influence the sensory symptoms seen in individuals with post-traumatic stress disorder | Single-subject ABA design | (4) Women with PTSD / Community | Quantitative / Single subject graphing and statistics. | The WTP may have efficacy as a possible new treatment for some clients with PTSD whose symptoms are related to difficulty with sensory modulation |
| Gimeno et al, 2019 (UK) | Explore preliminary effectiveness of the Cognitive Orientation to daily Occupational Performance (CO-OP) Approach in improving outcomes in childhood-onset hyperkinetic movement disorders following deep brain stimulation (DBS) | Randomized, multiple-baseline, Single-Case Experimental Design N-of-1 trial | (11) Participants with HBD / Community | Visual analysis. Statistical Analysis of Performance Change Between Baseline and Post-intervention. | Cognitive strategy use improved participant-selected functional goals in childhood-onset HMD, more than just practice during baseline. Preliminary effectiveness is shown when the intervention is delivered in clinical practice by different therapists in routine clinical settings |
| Gimeno et al, 2021 (UK) | Explore the feasibility, acceptability, and therapeutic potential of CO-OP for children with HMD | Single-case experimental design using baseline as n-of-1 | (10) Children and young people with hypertonic movement disorders / Community | Quantitative / Visual analysis and description, a range of quantitative statistical analyses, clinical significance of goal improvement. | A cognitive-based, task-oriented approach to support performance of personally relevant functional skills enabling participation is acceptable in childhood-onset HMD post-DBS. Preliminary efficacy to improve outcomes and proof of concept with CO-OP has been established in this population. |
| Go & Lee, 2016 (Korea) | Investigate the effect of an intensive sensorimotor stimulation programme on the motor function of chronic hemiparetic patients | AB single subject experimental design | (3) Chronic stroke patients whose sensory function was intact. / Not reported | Quantitative / Descriptive data analysis (p.3351) | The intensive sensorimotor stimulation programme for the upper extremity may be an efficacious method for improving the function of the affected limb of chronic stroke patients. |
| Golisz et al, 2018 (UK) | Identify changes in independence in activities of daily living following learning support using individualized everyday technologies | Double-baseline case study design | (3) Adult men with IDD / Community | Quantitative / Video analysis | Everyday technology applications and devices can be utilized together with a guided and structured client-centred approach and task-specific training with individuals with disability and learning difficulties. |
| Gontijo et al, 2016 (Brazil) | Describe an occupational therapy intervention aimed at sexual and reproductive health promotion in adolescents | Qualitative case study | (58) adolescents / Community | Qualitative / Descriptive statistics and thematic content analysis | Adolescents’ increased knowledge of sexual and reproductive health information immediately after the intervention. |
| Green et al, 2018 (USA) | Explore the effects of enhanced lighting on the function of people with an acquired visual deficit resulting from CVA in an Inpatient Rehab Facility | ABAB design - case report | (2) Adults/older adult with CVA and visual impairment / Inpatient | Quantitative / Analysis consisted of scores on the grooming section of the FIM®, times for each grooming task, and light metre readings | Both participants demonstrated improvements in function as measured by the FIM and in time to complete grooming tasks with enhanced lighting. |
| Gustafsson et al, 2016 (Australia) | Investigate the effectiveness of compression bandaging from the fingers to the axilla in reducing poststroke edema in the upper limb | ABA single-case research design | (5) People with post stroke u-L edema / Inpatient | Quantitative / Visual analysis | Compression bandaging of the entire upper limb may be an effective intervention option for reducing poststroke edema. Individual client differences support the need for careful monitoring of edema during and after compression bandaging. |
| Gustafsson et al, 2016b (Australia) | Explore whether Class 2 circular-knit compression gloves retain the reduction in edema after the application of compression bandaging to the affected upper limb in people with stroke | ABC single-case design | (4) individuals with post stroke U-L edema / Inpatient | Quantitative / Visual analysis. | The use of compression bandages seems effective in decreasing edema after stroke, but whether compression gloves can retain the reductions achieved remains uncertain. |
| Haines et al, 2018 (UK) | Explore the ways an occupational therapist supported people with PIMD to engage in activity at home. | Qualitative case study methodology | (1) Occupational therapy supporting people’s engagement in activity at home / Community | Qualitative / Inductive thematic analysis | Aspects of the organizational culture where people with PIMD live may impact on how they are supported to engage in activity. Inevitably time-limited interventions seem therefore best focused on creating and sustaining cultural change amongst those providing support. |
| Hejaz-Shirmard et al, 2020 (Iran) | Investigate the effects of sensory retraining on the light touch threshold of the hand, dexterity and upper limb motor function of chronic stroke survivors | Single-subject AB design | (5) Chronic stroke with right hemiplegia and 2 with left hemiplegia / Outpatient clinic | Quantitative / Visual analysis, nonparametric Mann-Whitney U test and, c-statistic | Adding sensory retraining to standard occupational therapy may improve the light touch threshold and enhance manual dexterity and upper limb motor function in chronic stroke survivors. |
| Henderson et al, 2020 (USA) | Determine the effects of LSVT BIG on occupational performance with an individual with PD | Single-case study design | (1) 73-year-old woman with PD / Not reported | Quantitative / Change scores calculated | Supports the use of LSVT BIG with an individual with PD to increase subjective and objective occupational performance outcomes. |
| Henning et al, 2016 (Australia) | Test the feasibility and preliminary effectiveness of a play-based intervention for children with ASD employing the above principles | Multiple case study design - ABA | (5) Parents and children with ASD / Outpatient clinic | Quantitative / Rasch analysis, line graph plotting, PND method to calculate effect size. | Demonstrated the preliminary feasibility and effectiveness of a play-based approach using therapist, peer and video modelling as well as parent involvement to improve the social play skills of children with ASD. |
| Hui et al, 2016 (Canada) | Explore the impact of school-based occupational therapy intervention on teachers’ classroom management self-efficacy and perceived performance/satisfaction in their management of students with disruptive behaviours | Multiple case replication design | (11) Elementary school teachers / School | Quantitative / person based analysis | Improvement in teachers’ perception of performance, satisfaction, and classroom management was seen. GAS showed clinically significant improvement. Improvements were sustained at 7 weeks follow-up. |
| Hunt et al, 2019 (Canada) | Determine the feasibility and effects of the CO-OP Approach for youth with persistent post-concussion symptoms. | Prospective case series | (3) Youth with post-concussion symptoms and parents / Community | Mixed methods / Descriptive analysis | The CO-OP Approach may be a treatment option for youth with persistent post-concussion symptoms. Clinically significant, positive changes were found in participants’ activity performance and satisfaction. |
| Hurst, 2017 (UK) | Understanding the relationship between theory and clinical practice and, specifically, how the CMOP has been integrated into working practices of occupational therapists | Qualitative case study | (11) Occupational therapy practitioners in one county who use the CMOP to underpin their practice / Community | Qualitative / Thematic analysis | This study identified that use of a single model; the CMOP actively encouraged practice development in this county and was a dynamic and multifaceted social process. The findings contribute theory building in occupational therapy practice |
| Hyett et al, 2017 (Canada) | Improve occupational therapists’ understanding of an approach to building community participation, through case study of a network of Canadian food security programmes | Qualitative instrumental case study | (1) A social network of food security programmes and initiatives across several provinces /Third sector | Qualitative / Thematic analysis | Occupational therapists can utilize an approach for building community participation that incorporates resource mobilization. Challenges of sustainability and social exclusion must be addressed |
| Hyett et al, 2019 (Canada) | Present a new conceptual framework for community-centred practice in occupational therapy | 2 sequential instrumental case studies | (2) a network of Canadian food security programmes and rural community banking initiative. /Third sector | Qualitative / Thematic analysis and cross case analysis | The outcome is a four-stage, occupation-focused, practice framework that will assist occupational therapists to conceptualize, understand, and apply a community-centred practice approach. |
| Park et al, 2020 (Korea) | Investigate the effects of family-centred early intervention on the quality of and skills in social interaction in infants with suspected autism spectrum disorder using a single-subject design | Single-subject design | (3) infants with ASD / Community | Quantitative / Visual analysis | Family-centred early intervention was confirmed to have a positive effect on the improvement of social interaction skills in infants with suspected autism spectrum disorder. |
| Iso et al, 2016 (Jaan) | Investigate whether a method of MP using an inverse video of a subject’s unaffected limb to complement the vividness of MI would be effective for improving affected upper limb function | ABA single-subject design | (1) 60-year-old man with left sided hemiparesis / Outpatient clinic | Quantitative / Visual analysis and the 2-standard deviation (2-SD) band method | This study suggested that an inverse video of a subject’s unaffected limb seems to be an effective tool for assistance of Motor Imagery in performing Mental Practice. |
| Johanson et al, 2019 (Sweden) | Illustrate the IES model and process in five cases, based on the perspectives of participants, employment specialists and on documents and memos | Multiple case design | (5) Individuals who had a depressive episode or bipolar and had been on long term sick leave / Community | Qualitative / Inductive content analysis. Within-case and cross case analysis | The IES is a way of promoting increased engagement in return to work, and learning new work-related behaviour and coping strategies. |
| Joyce & Warren, 2016 (Ireland) | Explore how participation in an allotment [gardening] group, facilitated through a mental health occupational therapy service, influences well-being | Qualitative case study methodology | (6) Three men and three women / Community | Qualitative Thematic analysis and visual analysis | Occupational therapy-led gardening group participation to be a therapeutic medium for normalizing, supportive, satisfying, skilled group work that achieved a positive influence on well-being, as revealed by the progression of participants in their personal and professional lives |
| Kassberg et al, 2016 (Sweden) | The aim of this study was to explore and describe how client-centred occupational therapy interventions may support and improve the ability to use everyday technology (ET) in work tasks in people with acquired brain injury (ABI). | Qualitative descriptive exploratory multiple case study | (3) Working age participants with ABI / Community | Mixed methods / Descriptive statistics, RASH analysis, qualitative descriptive summaries, pattern matching | Individual occupational-based intervention processes designed to support the ability to use ET in work tasks can enable improvements in performance of work tasks that include the use of ET. |
| Kearns Murphy & Sheil, 2021 (Ireland) *(linked reports; Kearns Murphy & Sheil, 2019;2019b)* | Evaluates the impact of individualized occupational therapy interventions to enable occupational engagement in the context of the transition to a home of one’s own in the community in Ireland | Longitudinal case study | (2) Men in residential facility with SMI / Community | Quantitative / Descriptive statistics and visual analysis | Provides initial evidence for intensive occupational therapy interventions delivered by OTAs under the supervision of an occupational therapist to address occupational performance and engagement in the context of the transition to one’s own home in the community in Ireland. |
| Kent et al, 2020  (Australia) | Explore the changes in play performance of children transitioning from a play dyad to triad and how these changes may influence future iterations of the PLF (ultimate guide to play language and friendship) | Multiple case study | (5) children with ASD each playing with two known TD peers / Community | Quantitative / Rasch analysis plotting interval level measures scores (visual analysis) | Four of the five children with ASD generalized their play performance from the dyad to the triad social environment. However, the triad intervention did not demonstrate improvements in play performance. |
| Khoshbakht et al, 2021 (Iran) | Investigate the effects of somatosensory interventions on upper extremity sensory and motor functions in spastic hemiplegic children | Single subject | (3) 2 boys 1 girl with spastic hemiplegia / Outpatient clinic | Quantitative /Visual analysis, statistical 2 standard deviation | The 4-week sensory intervention programme used in this study could serve as an effective supplementary treatment alongside common motor interventions for children and young people with hemiplegic cerebral palsy being treated in occupational therapy clinics. |
| Kim & Kim, 2018 (Korea) | Treat dysphagia in a newborn baby with cri du chat syndrome using an oral stimulation intervention and to examine its effects | Single-subject AB design | (1) 2-week premature baby with cri du chat in NICU / Inpatient | Quantitative / Frequency analysis, comparatively analysed using visual graphs and descriptive statistics | The oral stimulation intervention provided prior to feeding resulted in highly positive effects, including induced normal development of the baby, stimulation of his transition from the NG feeding tube to bottle feeding, increased oxygen saturation, and a shortened hospital stay. |
| Lamarre et al, 2020 (Canada) | Explore the use of OPC in this assisted living | Single instrumental case study | (1) Resident in assisted living / Community | Qualitative / Narrative analysis, framework method (thematic analysis) | Despite considerable physical and cognitive challenges, application of OPC allowed the participant to engage in a highly valued occupation in a desired community context. |
| Lawson et al, 2017 (USA) | Investigate the utility of a mobile app to improve motor control for stroke survivors by examining changes in motor ability and participation | Multiple case study | (6) Adults with stroke / Community | Mixed methods  Changes in pre and post tests | The utility of this form of motor training is promising in its potential to meet the parameters of motor learning, promote neuroplasticity, and be engaging and easily accessible to multiple populations, such as those in underserved areas. |
| Lorenzo et al, 2019 (South Africa) | investigate how non-governmental organizations (NGOs) facilitate the participation of disabled youth and young parents of disabled children in sport and other free-time activities to enable their transition to livelihoods development | Exploratory intrinsic case study | (1) The organizational capacity of not-for-profit organizations  (NPOs) and NGOs to facilitate the skills development for decent work through participation in sport and other free-time activities. / Community | Qualitative / Deductive analysis of transcripts, cross analysis of transcripts. | The centrality of occupational therapists in NGOs to advocate with families for accessible and affordable communication, information and transport systems for youth with disabilities at a local municipal level will improve access to occupations for inclusive livelihoods development for all youth. |
| McCourt & Casey, 2016 (UK) | Evaluate the readiness of children (aged 4–9 years), who already use a NHS powered indoor wheelchair to drive an EPIOC (electrical powered indoor/outdoor chair test) under supervision of an adult caregiver | Case study design | (3) Children using powered wheelchair / Community | Quantitative / EPIOC assessment score | Regional Northern Ireland criteria relating to electrically powered indoor/outdoor chair provision for children should be revised in order to consider an individual needs assessment, rather than imposing an age restriction. |
| Metcalfe et al, 2019 (Canada) | Explore whether the LSVT BIG programme could lead to improved performance in client-identified occupations and decreased impairment late poststroke | Single-case experimental study | (2) Adults who experienced stroke / Community | Mixed methods / Visual analysis and medians from ordinal scales and split middle method, Interview responses were summarized with quotations provided | Performance improved on either self-assessment or blinded rater assessment for all but one activity LSVT BIG is a promising intervention to improve occupational performance. |
| Mohammadi et al, 2017 (Iran) | Investigate the effects of play‑based occupational therapy on pain, anxiety, and fatigue in hospitalized children with cancer who were receiving chemotherapy | Single-subject study | (2) Children with cancer / Inpatient | Quantitative / Visual analysis and line charting | Play‑based occupational therapy can be effective in improving pain, anxiety, and fatigue levels in hospitalized children with cancer receiving chemotherapy |
| Nilsson et al, 2020 (Sweden) | Explore changes in work potential and work performance for ten people who worked before their stroke whilst participating in the programme and to describe measures performed by the occupational therapists (OTs) to enhance work potential and work performance during the programme | Multiple case study | (10) People who had experienced mild/moderate stroke. / Outpatient clinic | Mixed methods / Descriptive statistics and Content analysis | The ReWork-Stroke programme seems promising for promoting changes in work potential, work performance, and return to paid work. |
| Page et al, 2016 (USA) | Determine the impact of repetitive task-specific practice (RTP) integrating electrical stimulation and behavioural supports on upper extremity (UE) impairment, gross motor dexterity and paretic UE amount and quality of use in chronic stroke survivors exhibiting moderate stable UE deficits | Case series with 3 month follow-up | (6) Persons who experienced a stroke >12 months before enrollment and exhibiting chronic, moderate stable UE impairment / Outpatient clinic | Quantitative / Wilcoxon signed ranks test | The addition of behavioural supports to an already validated RTP regimen significantly increases paretic UE use and function. |
| Pashazadeh Azari et al, 2020 (Iran) | Investigate the feasibility and effectiveness of Contextual Intervention Adapted For Autism Spectrum Disorders (CI-ASD) in occupational performance of developing ASD children and their mothers’ self-efficacy | Single-subject quasi-experimental study with ABA design | (2) Mothers of children with sensory processing difficulties / Outpatient clinic | Mixed methods / Visual analysis | CI-ASD is an acceptable intervention for children with ASD and their families |
| Peny-Dahlstrand et al, 2020 (Sweden) | Investigate whether the CO-OP approach is feasible for and potentially beneficial to adolescents and young adults with CP or SB in Sweden by analysing four areas of feasibility | Exploratory multiple case study embedded design | (10) Persons with CP or spina bifida / Community | Mixed methods / Interpretive analysis performed in Framework analysis. | The Cognitive Orientation to daily Occupational Performance is a feasible approach for adolescents and young adults with spina bifida and with cerebral palsy. |
| Peruzzolo et al, 2018 (Brazil) | Analyse the effectiveness of a Hypothesis of Psychomotor Functioning (HPF) for treatment of the premature babies | Qualitative, longitudinal, and clinical almost experimental single-case study | (1) Premature baby / Not reported | Qualitative / Yin’s (2001) ‘Logical Model of Program’, a combination of matching techniques to found pattern and time series analysis | The HPF interprets the baby psychomotor process based on its construction, allowing a PI that can transform a psychomotor symptom into a structuring motion. |
| Peters et al, 2020 (USA) | Pilot a screening, evaluation, and intervention protocol of occupational therapy in an equine environment and assess preliminary effects on occupational performance goals, behaviour, and social functioning of youth with autism | Multiple baseline single-case experimental design | (6) Youth with autism / Community | Quantitative / Visual analysis and descriptive statistical analysis | Participants improved in occupational performance goals, social motivation, and communication; four demonstrated decreased irritability and hyperactivity. |
| Pingale et al, 2020 (USA) | Investigate the effect of sensory diets on children’s sensory processing, psychosocial skills, and classroom engagement behaviours in the school environment | Single-subject ABA design | (3) Children with SPD / School | Quantitative / Visual analysis | Sensory diets administered in brief sessions in the school day appear to be effective in improving children’s sensory processing, psychosocial, and classroom engagement behaviours and may have a continued beneficial effect. |
| Pressiner, 2016 (USA) | Explore how fatigue affected identity from the perspectives of people with multiple sclerosis (MS) | Qualitative case study | (7) individuals with MS / Community | Qualitative / Constant comparative method used to derive themes including using open and axial coding | Participants changed how they performed daily activities, which changed self-perception. Discussion in the fatigue management group helped individuals reconceptualize their situations. |
| Pretorious, 2018 (South Africa) | Explore and describe the contribution of occupational therapy in the holistic management of TAS | Single descriptive case study | (1) Child with TAS. The case will be bound to this one specific individual and the context within she exists. / Community | Mixed methods / Content analysis, document analysis and direct observation | By looking at this case holistically and considering the person, environment and what occupations are important and meaningful, an occupational therapist can contribute greatly to the overall quality of life, wellness and satisfaction of a child with TAS. |
| Proffitt et al, 2018 | Determine whether participation in the LSVT BIG programme had an effect for a client with stroke and whether it was feasible to deliver the home exercises using a game-based virtual reality platform | Case report | (1) 56-year-old woman with stroke / Outpatient clinic | Mixed methods / Statistical and thematic analysis | The intensity of the LSVT BIG intervention coupled with the integrated neuroplasticity principles and the game based delivery of the home programme led to the participant making gains beyond what was expected. |
| Provancha-Romeo et al, 2019 | Explore the use of mind-body interventions as a tool for use by occupational therapists (OT) to improve health and occupational performance | Exploratory case study | (1) 57-year-old female / Inpatient | Quantitative / Percent change was calculated for each variable | Mind-body interventions may potentially be useful to reduce stress and anxiety related symptoms, both frequently experienced by patients in the ICU. |
| Radomski et al, 2018 (USA) | Evaluate the feasibility of an intervention combining metacognitive strategy instruction (MSI) with training in implementation intentions for adults with mild traumatic brain injury (mTBI) | Pretest–posttest case study design | (2) Women with mTBI / Outpatient clinic | Quantitative / Not reported | Cognitive intervention that combines MSI with training in implementation intentions may enhance individualized goal setting and attainment in occupational therapy for clients with mTBI and can be considered as a treatment option. |
| Ribiero et al, 2019 (USA) | Understand the intervention of Occupational Therapy in the Therapeutic Community ‘Clínica do Outeiro – Portugal’ | Descriptive exploratory case study research | (1) Rehabilitation of individuals with substance use disorders / Community | Qualitative / Content analysis | Occupational Therapy plays a preponderant role in the process of daily reconstruction of individuals with substance use disorders. It is a contribution for the reflection of politicians, administrators and other professionals about the need of Occupational Therapists in these rehabilitation contexts |
| Ribeiro et al, 2017 (Portugal) | Understand the perception of clients and OT on intervention priorities, trying to determine whether there is compliance between the views | Descriptive and exploratory case study | (6) 3 clients and 3 occupational therapists of the department of psychiatry and mental health of the hospital Infante D Pedro / Inpatient | Qualitative / Content analysis | OT and clients have differing views regarding intervention and its priorities. It was found that clients are not fully satisfied for not being allowed to work for individual goals. |
| Rowe & Neville, 2018 (USA) | Explore the effectiveness of a Task Oriented Training and Evaluation at Home (TOTE Home) programme completed by people with subacute stroke, and whether effects persisted 1 month after this training | Single-subject design | (4) Individuals with stroke / Community | Quantitative / Graphic analysis and visual inspection | Four participants completed TOTE Home and each demonstrated improvement in movement and confidence in function. This study contributes to the effectiveness of TOT and is a model easily replicated in practice. |
| Soeker & Pape, 2019 (Africa) *(linked report; Soeker, 2016)* | Explore and describe the experiences of individuals with TBI regarding returning to work through the use of the Model of Occupational Self-Efficacy | Qualitative exploratory multiple case study | (10) Individuals with TBI / Community | Qualitative Explanation building, interviews were coded, categorized, and placed into themes | MOOSE is a useful model to use in retraining work skills to an individual with brain injuries. |
| Sonday & Gretschel, 2016 (South Africa) | Explore the impact of powered mobility on the exploratory play of two children with physical disabilities | Qualitative, collective case study design | (2) Children using powered wheelchairs / Community | Qualitative / Inductive within and cross case analysis (categories and themes) | Provision of powered mobility is a key contributor promoting the participation of physically disabled children in exploratory play |
| SØrlie et al, 2020 (UK) | Report on the preliminary evidence for the utility of the PPR Profile used with individuals with eating disorders with the support of an occupational therapist | Descriptive case study | (6) Women with eating disorder attending the occupation matters group / Community | Qualitative / Inductive thematic content analysis | Provides supportive evidence for the use of an occupation-focused assessment with eating disorders populations |
| Stickley & Hall, 2017 (UK) | Examine the compatibility of occupational therapy practice and a social enterprise environment, within the UK | Case study | (8) Social enterprises / Community | Qualitative / Thematic analysis | Demonstrated that occupational therapy philosophy and principles can work effectively within a social enterprise environment |
| Stier et al, 2017 (Canada) | Examine parent-reported change in the functional performance of four school-aged children with wheeled mobility needs who had used a new adaptive seating system for 6 weeks | Collective case study | (4) Parents and children with CP/GDD who got issued a new powered wheelchair / Community | Mixed methods / Minimum detectable change scores for the total FIATS-AS and Thematic content analysis | The FIATS-AS detected overall functional gain in one family, and both gains and losses in 2–7 dimensions for all families. |
| Suarez & Bush, 2020 (USA) | Investigate whether the JRCFP was effective at increasing the number of foods that the children accepted as part of their regular diet | Single-subject multiple baseline across participants | (7) Children with food selectivity / Outpatient clinic | Quantitative / Visual analysis, Percentage of Nonoverlapping Data (PND), Wilcoxon Matched-Pairs Signed-Rank | Five out of seven children accepted significantly more foods after a treatment latency period. Understanding a child’s sensory profile, and the presence of SOR, may assist with matching food selectivity treatment to the child’s needs. |
| Suder et al, 2016 (USA) | Determine whether children with cerebral palsy (CP) can adhere to and gain motor skills from a home-based treatment of hand therapy video games in combination with contralaterally controlled electrical stimulation (CCFES) | Case series pilot | (Not reported) children with CP and hemiparesis / Not reported | Mixed methods / Descriptive statistics for cross case analysis and paired t tests | Initial data demonstrates a positive trend toward improved fine motor and improved occupational engagement. demonstrates potential for a viable and important discovery in technology for occupational therapists to use for the treatment of children with hemiplegic CP. |
| Teixeira & Alves, 2021 (Brazil) | Assess the AT requirements of wheelchair rugby players, identify the psychosocial factors that contribute to their performance and AT use and assess user satisfaction with the recommended and implemented AT | Descriptive explanatory case study with a quantitative approach | (4) wheelchair rugby players from the Lobos Vermelhos (Red Wolves) team of the Special Physical Education Training Centre Association (CETEFE), / Not reported | Quantitative / Statistical (frequency) analysis | The AT improved athletic performance and increased the overall satisfaction score from 1.6 to 4.8 after intervention. The factors considered to determine AT success were safety, comfort and effectiveness, and the positive psychosocial factors identified were support from family and friends and sufficient income to participate in the sport. |
| Umeda, 2017 (USA) | Investigate family and organization experiences and impacts of a sensory friendly theatre programme developed with organization-level occupational therapy consultation. A secondary aim was to investigate organization perspectives on the sensory friendly programme development process and partnership with the occupational therapy consultant | Qualitative case study methodology | (7) Parents of children who attended the sensory friendly theatre / Third sector | Qualitative / Inductive thematic analysis and case comparison to identify patterns and differences | Successful community cultural arts participation is a valued outcome for parents of children with disabilities. Utilizing organization-level occupational therapy consultation to support access initiatives and build capacity within cultural arts organizations is a promising new approach in occupational therapy service delivery. |
| Verikios et al, 2016 (Australia) | Investigate the physical, psychosocial and functional impact of the TAPit on an individual living with spinal cord injury | Single mixed methods case study design | (1) 50-year-old woman / Community | Mixed methods / Descriptive statistics. Within case | Provides the first evidence about the effectiveness of the TAPit for people with spinal cord injury, including participant perspectives on its use. Demonstrates its potential to support people to be able to participate in meaningful activities and occupations and that the provision of suitable adaptations and apps are the key to its success. |
| Wallis et al 2018 (New Zealand) | Explored the effectiveness of a 6-week sensory modulation intervention for reducing anxiety and improving occupational participation | Mixed methods multiple repeated measures single-case design | (4) Participants meeting inclusion criteria / Community | Mixed methods / Statistical analysis and qualitative descriptive methods | Support the use of sensory modulation for the management of anxiety within a community mental health setting. Also provides preliminary evidence that sensory modulation is a cost-limited practical intervention that builds resilience, self-regulation, and can be accessed in a timely manner |
| Weichbrodt et al, 2018 (Sweden) | Evaluate whether treatment of boys with Duchenne muscular dystrophy using hand orthoses could benefit joint mobility, grip strength, or fine motor function | Single-subject design | (8) Boys with Duchenne muscular dystrophy / Outpatient clinic | Quantitative / Descriptive comparison and visualized in diagrams. Separate case analysis and composite analysis | Hand orthoses can delay development of contractures and preserve hand function and give prerequisites for independence. The occupational therapists should measure wrist joint mobility regularly to be able to find the right time for intervention with hand orthoses in this progressive disorder. |
| Wilkes-Gillan et al, 2017 (Australia) | Investigate child outcomes and mothers’ perspectives following technology-based intervention sessions aimed at improving children’s social play skills | Mixed methods multiple case study | (5) Children with ADHD / Community | Mixed methods / Descriptive and visual analysis by case and group | Adds to emerging evidence that suggests video-modelling may be an engaging, feasible and beneficial intervention tool for improving the social difficulties of children with ADHD. |
| Wolfhope & Hudkins, 2016 (USA) | Identify if use of iLS Dreampad mini (DPM) and FitBit Flex (FBF) increased quality of sleep (QOS) and overall function in daily occupation in children with autism spectrum disorder | Pre-experimental case study (OXO) | (2) Children in the local area with ASD / Community | Mixed methods / Descriptive statistics & thematic analysis. inferential statistics in progress | iLS mini is an effective tool to increase functional participation in daily occupation, specifically sleep preparation and participation by increasing quality of sleep, mood regulation and attention and focus for children with autism spectrum disorder |
| Yoshihiro & Ito, 2017 (Japan) | Examine the effect of passive limb activation by functional electrical stimulation (FES) on wheelchair driving for patients with USN | Single-subject ABA design | (2) Stroke patients with Unilateral spatial neglect / Inpatient | Quantitative / Visual analysis and binomial tests | Results suggest that passive limb activation by FES could enhance the effects of the intervention on ADL. |
