# Supplementary material for: Exploring the contribution of case study research to the evidence base for occupational therapy: a scoping review
Source: Syst Rev. 2023 Jul 31;12:132. doi: 10.1186/s13643-023-02292-4 (PMC10388505; doi:10.1186/s13643-023-02292-4)
Supplement: Supplementary file 6 — Additional file 6. Characteristics of included non-empirical papers. [file 13643_2023_2292_MOESM6_ESM.docx]

| Study (country) | Aim | Key findings | Definition of methodology | Reported Strengths of methodology | Reported limitations of methodology |
| --- | --- | --- | --- | --- | --- |
| Carey, 2020 (UK) | To conduct a descriptive analysis of contemporary use of case study research in occupational therapy based on selected articles using this methodology | 32 papers synthesized; global uptake of the methodology; multiple data methods used to triangulate findings.  Settings were wide and varied both in clinical setting, condition and age range of service users.  46% of explanatory studies focus on the ‘how’ questions; 53% of exploratory studies focus upon the ‘why’ questions.  60% investigated a specific occupational therapy service. 40% were interventions related to either service users or students. | In-depth, multifaceted exploration of complex issues in their real-life settings.  Capacity to describe in-depth a clinical intervention, understand effects of a service policy or explore professional attitudes.  Qualitative, quantitative and mixed methods | An alternative which more reflects the essence of the profession.  Potential to describe the person, the occupation and the environment encapsulating the complexity of subjectivity within it.  It enables complexity of a multitude of elements to be broken down, investigated and analysed. | Cannot be considered as generalizable in the context that positivist research define generalization. It is for the researcher to convey to the reader thorough understanding and interpretation of the case; it is through such depth of understanding that links, comparisons and contrasts can be made within relevance and significance |
| Hercegovac et al, 2020 (Australia) | To explore how QCSM has contributed to occupational  therapy knowledge | 27 studies included; Diverse range of practice settings; Triangulation evident in all studies through use of multiple data collection methods.  Studies examined;  outcomes of occupational therapy interventions (*n*=12).  Concepts relating to occupational science or theoretical concepts (*n*=9)  Exploration of the clinical reasoning (*n*=4)  Exploration of novel research methods (*n*=2).  Lack of consistency when referencing seminal authors; Only five studies gave adequate definition of the case | Phenomenon of interest that is situated in a natural, real-life context, where the boundaries between the phenomenon and context are blurred.  Defined by the number of cases and by their purpose. | Examining cases in real-life contexts compliments the holistic perspective that occupational therapists use in practice  Its flexibility to capture the complexity of the phenomenon under study and consider the context in which it is situated  Suited to exploring a variety of complex research questions | Criticisms over a lack of rigour in its research methods and small sample sizes, i.e. lack of generalizability  Interpreted from different theoretical perspectives, which may have contributed to a lack of consistency in its application |
| Jonasdottir et al, 2018 (Canada) | Part 1 provides a brief overview of what case study methodology is; and part 2 presents an integrated review on  how case study has been used for the study of occupation | 18 studies included; various terms to define the type of case study  Most studies were exploratory (*N* = 12)  Case and boundary not always clear. Most common cases were individuals (*N* = 11). Other cases were couples (*N* = 2), a group (*N* = 1) and an organization (*N* = 1)  Dominant data collection methods were interviews (*N* = 18) and observations (*N* = 13). Various terms were used for data analysis | An in-depth study of a bounded phenomenon (a case) in its real-life context. Suitable to answer ‘how’ and ‘why’ questions where the focus is on processes or ‘what’ questions that are intended to understand the case. Includes rich or thick description of both the case and its context in order to take the reader into the case situation. | useful ‘to understand the complexities’ of occupation, as a phenomenon embedded in the messiness of people’s everyday lives  Flexible regarding the paradigmatic stance of the researcher | vague and inconsistent use of the term ‘case study’ and divergent publications on the topic  Challenges adhering to word limits in academic journals whilst simultaneously presenting thick description of the case in its context |
| Lane et al, 2017 (USA) | To highlight the similarities between the mission of occupational therapy and SCEDs. | Recommendations for designing single-case studies are provided;  Complex concepts should be measured through careful identification of related, observable behaviours  Being familiar with SCEDs, along with behaviours of interest ensures a research question can be adequately answered  Practitioners and researchers are encouraged to consider guidelines for conducting methodologically rigourous studies, e.g. SCRIBE  The most beneficial information for the field is generated when appropriate designs are used, contemporary guidelines for rigour are followed, positive outcomes occur, and researchers replicate findings within and across participants | An SCED research approach involves repeated measurement of observable behaviours. SCEDs include several designs that allow practitioners and re-searchers to formatively evaluate the impact of an intervention with a small number of participants. Not the same as case studies which are part of a qualitative research approach. Extending findings beyond an individual SCED is a matter of replication within a study and across studies. | SCEDs are well suited for evaluating interventions conducted in applied settings where practitioners provide therapeutic services, such as community clinics, schools and home.  SCEDs allow researchers  to evaluate the impact of an intervention by collecting data on a day-by-day or session-by-session basis.  Allows for changes or modifications if an intervention is not working as planned which corresponds well with ethical clinical practice | The extent to which findings from SCEDs are generalizable is oftentimes criticized |
