# Supplementary material for: Exploring the contribution of case study research to the evidence base for occupational therapy: a scoping review
Source: Syst Rev. 2023 Jul 31;12:132. doi: 10.1186/s13643-023-02292-4 (PMC10388505; doi:10.1186/s13643-023-02292-4)
Supplement: Supplementary file 7 — Additional file 7. Studies ineligible following full-text review post 2016. [file 13643_2023_2292_MOESM7_ESM.docx]

# Additional File 2: Studies ineligible following full-text review post 2016

*Exclusion Reason: Concept – not case study research*

| Authors | Title |
| --- | --- |
| Ciucurel M, Iconaru EI, Ciucrel C, 2016 | A case study of occupatioanl therapy application in a patient with depression and hypoacusis |
| Bresi-Ando J, 2017 | Case study: Neurological rehabilitation |
| All answers Ltd, 2018 | Integrated Occupational Therapy Practice Case Study |
| AABOL R. & SVEEN U, 2016. | Occupational performance and return-to-work after mild traumatic brain injury-A case report |
| ABLORT-MORGAN, C., ALLORTO, N.L. & RODE, H., 2016. | Rehabilitation of a bilateral upper limb amputee in a resource restricted burn service. |
| ALTERIO, C 2018. | Use of a Case Study Method to Explore Sensory Processing Characteristics of Children With Complex Regional Pain Syndrome |
| ALVIAL, P., BRAVO, G., BUSTOS, M.P., MORENO, G., ALFARO, R., CANCINO, R. & ZAGAL, J.C., 2018. | Quantitative functional evaluation of a 3D-printed silicone-embedded prosthesis for partial hand amputation: A case report. |
| ANABY, D., GORTER, J., LEVIN, M., AVERY, L., TEPLICKY, R., CORMIER, I., TURNER, L., COULTER, J. & HANES, J., 2019. | The impact of participation-based interventions on body functions among youth with physical disabilities. |
| ANDERSON, R. & REZNICK, H. 2016 | The power of occupational engagement: Norms story. |
| ATHANASOPOULOS, K.G., PAPANASTASSIOU, I.D., DRINIS, I., GROUMAS, N., GEROCHRISTOU, M.A. & PETROPOULOU, K., 2019. | A patient with guillain-barre syndrome and late recovery after 1 year. |
| BANUMATHE, K.R., GURUPRASAD, V., KARTHIK RAO, N. & SUKUMAR, S., 2016. | Extended occupational therapy based rehabilitation for a young client following head injury - a case report. |
| BERG, C., KING, A. & EDWARDS, D.F., 2018. | Mentoring Program for Young Adults with Sickle Cell Disease. Occupational Therapy in Health Care. |
| BIGONI, M., BAUDO, S., CIMOLIN, V., CAU, N., GALLI, M., PIANTA, L., TACCHINI, E., CAPODAGLIO, P. & MAURO, A., 2016. | Does kinematics add meaningful information to clinical assessment in post-stroke upper limb rehabilitation? A case report. |
| BOOTH, V., HARWOOD, R.H., HOOD-MOORE, V., BRAMLEY, T., HANCOX, J.E., ROBERTSON, K., HALL, J., VAN DER WARDT, V. & LOGAN, P.A., 2018. | Promoting activity, independence and stability in early dementia and mild cognitive impairment (PrAISED): development of an intervention for people with mild cognitive impairment and dementia. Clinical |
| BORGESTIG, M., SANDQVIST, J., AHLSTEN, G., FALKMER, T. & HEMMINGSSON, H., 2017. | Gaze-based assistive technology in daily activities in children with severe physical impairments-An intervention study. |
| BORNHEIM, S., MAQUET, P., CROISIER, J.L., CRIELAARD, J.M. & KAUX, J.F., 2018. | Motor cortex Transcranial Direct Current Stimulation (tDCS) improves acute stroke visuo-spatial neglect: A series of four case reports. |
| BORNMAN, J. & LOUW, B., 2019. | Personal Commitment Statements: Encouraging the Clinical Application of Continuing Professional Development Events for Health Practitioners in Low- and Middle-Income Countries. |
| BRANCH, M.S., 2019. | Acute rehabilitation management of diabetic muscle infarction: A case report. |
| CAMERON, D., CRAIG, T., EDWARDS, B., MISSIUNA, C., SCHWELLNUS, H. & POLATAJKO, H.J., 2017. | Cognitive Orientation to daily Occupational Performance (CO-OP): A New Approach for Children with Cerebral Palsy. |
| CAMPBELL, D., 2018. | Occupational therapy in action: A library of case studies. |
| CHAVIANO, K.N., ESTLER, P., SRINIVASAN, R. & EARLY, D., 2019. | Functional outcomes in a 3-year-old with Guillain-Barre syndrome using a multimodal therapeutic approach across a continuum of care: A case report. |
| CIAMPA, M.A. & LISCHINSKY, A., 2016. | Specific vocational and occupational rehabilitation programme with a patient with cerebellum injury: Case report. |
| CHARNEY, L., AIMES, J., APGAR, L., BUDAY, A., CALVERLEY, N., COTTA, N., KEARNEY, C., REDMOND, A. & SPIRES, T.April 19 to April 22, 2018 | Using Single-Subject Design and Goal Attainment Scaling to Measure Improvement in Social Participation in Children With Autism Spectrum Disorder |
| COLEOPY, D. & N, D.,La Perrelle, 2018. | Simulator assessment for obtaining medical clearance for driving following severe traumatic brain injury: A case study example. |
| COLLIER, L. & POOL, J., 2016. | Special Issue: Dimensions of dementia. |
| CONGE, H.A. & JARACZEWSKA, E., 1994. | Case report. Rehabilitation of spinal cord sarcoidosis. |
| CREEK, J. & COOK, S., 2017. | Learning from the margins: Enabling effective occupational therapy. |
| CROSS, A.L. & KHURANA, S.R., 2017. | Functional outcomes of patients after bilateral lung transplant: A case series. |
| CUNNINGHAM, R. & VALASEK, S., 2019. | Occupational Therapy Interventions for Urinary Dysfunction in Primary Care: A Case Series. |
| DAHI, A.S.A., 2019. | Isometric and Resistance Exercises Improved the Functional Ability of a Patient with Guillain-Barre Syndrome: A Case Study. |
| DERAKHSHANRAD, S.A. & PIVEN, E., 2019. | Modification of the training environment to improve functional performance using blacklight conditions: A case study of a child with autism. |
| DERBICH, J., 2016. | Bathroom adaptation to meet the expectations of the client suffering from Multiple Sclerosis (MS) as an example of occupational therapy process. |
| DIMEO, S.E.&Baxter, Mary F. 2017. | Matching students to augmentative and alternative communication: A survey of trends and a case study. |
| DUDLEY, D.R., KNARR, B.A., SIU, K., PECK, J., RICKS, B. & ZUNIGA, J.M., 2019. | Testing of a 3D printed hand exoskeleton for an individual with stroke: a case study. |
| DUNAWAY, S., DEZSI, D.B., PERKINS, J., TRAN, D. & NAFT, J., 2017. | Case Report on the Use of a Custom Myoelectric Elbow-Wrist-Hand Orthosis for the Remediation of Upper Extremity Paresis and Loss of Function in Chronic Stroke. |
| DUNNE, B.D., ROBINSON, K. & PETTIGREW, J., 2018. | Irish Journal of Occupational Therapy. 46(1), pp.31-45. |
| DUROCHER, E., KINSELLA, E.A., MCCORQUODALE, L. & PHELAN, S., 2016. | Ethical Tensions Related to Systemic Constraints. |
| EKELMAN, B.A., ALLISON, D.L., DUVNJAK, D., DIMARINO, D.R., JODZIO, J. & IANNARELLI, P.V., 2017. | A wellness program for men with spinal cord injury: Participation and meaning. |
| ERLER, K., 2018. | Acute Care Ethics Case Example: Sam. |
| FINLEY, W.P. & VAN LEW, S., 2018. | Occupational Therapy for Nonoperative Four-Part Proximal Humerus Fracture: A Case Report |
| FITZGERALD, M., SMITH, A.K., REHMAN, N. & TAYLOR, M., 2017. | Role Emerging Placements in Undergraduate Occupational Therapy Training: A Case Study. |
| FLUET, G.G., PATEL, J., QIU, Q., YAROSSI, M., MASSOOD, S., ADAMOVICH, S.V., TUNIK, E. & MERIANS, A.S., 2017. | Motor skill changes and neurophysiologic adaptation to recovery-oriented virtual rehabilitation of hand function in a person with subacute stroke: a case study. |
| GIMENO, H., POLATAJKO, H.J., CORNELIUS, V., LIN, J. & BROWN, R.G., 2018. | Protocol for N-of-1 trials proof of concept for rehabilitation of childhood-onset dystonia: Study 1. |
| GORDON, C., LAPOINTE, J., …, J MACLACHLAN - WORLD,FEDERATION OF & 2016, | U. A case study of a national association's actions to advance the integration of occupational justice and human rights in occupational therapy. |
| GRENIER, M. & SHANKLAND, B., 2020. | The use of static progressive and serial static orthoses in the management of elbow contractures after complex fracture dislocation injuries: A pediatric case study. |
| GRIFFIN, J., BASSINGTHWAIGHTE, L. & FLEMING, J., 2018. | Driving remediation following acquired brain injury: A case study series. |
| KIM, W.B., LEE, S.Y., KIM, B.R. & KIM, Y.J., 2019. | Rehabilitation of neuromyelitis optica: Two CARE-compliant case reports. |
| GUSTAFSSON, S., FALK, C., TILLMAN, S., HOLTZ, L. & LINDAHL, L., 2018. | Life filming as a means of participatory approach together with older community-dwelling persons regarding their local environment. |
| HAMM, J. & MONEY, A., 2019. | Guidetomeasure-OT: A mobile 3D application to improve the accuracy, consistency, and efficiency of clinician-led home-based falls-risk assessments. |
| HILLER, A., MADHU, S. & ERLANDSON, E., 2017. | Factor v Leiden mutation in a young adult male with ischemic stroke: A case study. |
| HIRTH, M.J., HOWELL, J.W. & O'BRIEN, L., 2017. | Two case reports-Use of relative motion orthoses to manage extensor tendon zones III and IV and sagittal band injuries in adjacent fingers. |
| HYEJIN, L. & BORAM, L., 2017. | The effect of combined upper limb robot treatment in patient with spinal cord injury: Case report. |
| IMHOFF, S., LAVALLIERE, M., GERMAIN-ROBITAILLE, M., TEASDALE, N. & FAIT, P., 2017. | Training driving ability in a traumatic brain-injured individual using a driving simulator: A case report. |
| HUBBUCK, M., FANG, L., MCANDREW, R. & KASKUTAS, V., 2019. | Occupation-Based Upper Extremity Rehabilitation: A Case Study. |
| IYER, A.S., 2018. | Occupational therapy management of the cognitive-perceptual deficits in a child with epilepsy-a case report. |
| IYER, A.S., JAHAGIRDAR, S. & KULKARNI, C., 2018. | Client centered occupational therapy in neurodegenerative conditions-case series. |
| JAMES, K., 2016. | Occupational therapists in Emergency Departments. |
| JAMWAL, R., CALLAWAY, L., WINKLER, D., FARNWORTH, L. & TATE, R., 2018. | Evaluating the Use of Smart Home Technology by People With Brain Impairment: Protocol for a Single-Case Experimental Design. |
| JANSON, R., BURKHART, K., FIRCHAU, C., HICKS, K., PITTMAN, M., YOPPS, M., HATFIELD, S. & GARABRANT, A., 2020. | Three-dimensional printed assistive devices for addressing occupational performance issues of the hand: A case report. |
| KORNAFEL, T., TSAO, E.Y., SABELHAUS, E., SURGES, L. & APKON, S.D., 2017. | Physical and Occupational Therapy for a Teenager with Acute Flaccid Myelitis: A Case Report. |
| KRINGLE, E.A., CAMPBELL, G., MCCUE, M., BARONE GIBBS, B., TERHORST, L. & SKIDMORE, E.R., 2019. | Development and feasibility of a sedentary behavior intervention for stroke: a case series. |
| SIMPSON, S., 2016. | Use of learning theories with an adult autistic client - a case study. |
| SINCLAIR, C., MEREDITH, P. & STRONG, J., 2018. | Case formulation in persistent pain in children and adolescents: The application of the nonlinear dynamic systems perspective. |
| SMITH, A. & MATTINGLY, R., 2017. | Alternatives to currently accepted feeding interventions when medical conditions restrict therapy: A case study. |
| LATHAM, C., 2019. | A CASE STUDY ANALYSIS OF THE SCOPE OF THE OCCUPATIONAL THERAPY ROLE IN A CRITICAL CARE UNIT. |
| LAVER, K. & WILKES‐GILLAN, S., 2018. | Video modelling interventions improve social communication skills for individuals with Autism Spectrum Disorder. |
| MARX, K.A., SCOTT, J.B., PIERSOL, C.V. & GITLIN, L.N., 2019. | Tailored Activities to Reduce Neuropsychiatric Behaviors in PersonsWith Dementia: Case Report. |
| MEHROTRA, S. & DSOUZA, S.A., 2017. | Meeting community needs through collaborative partnerships: A case study of a novel awareness program for learning disability using creative art forms. |
| MEISSNER, R.J., FERGUSON, J., OTTO, C., GRETSCHEL, P. & RAMUGONDO, E., 2017. | A play-informed, caregiver-implemented, home-based intervention for HIV-positive children and their families living in low-income conditions in South Africa. |
| SNYDER, J. & SALIM, S., 2018. | Poster 281: Unique Adjustments in Physical and Occupational Therapies Enhance Recovery from Spinal Cord Injury in Two Patients with Achondroplasia: A Case Report. |
| MIN-SOOK KIM, SO-YEON PARK, WON-MEE JEONG, SOO-HYUN PARK & KYOUNG-YOUNG PARK, 2018. | A Case Study of an Occupational Activity Program Following the Great East Japan Earthquake and Tsunami and the Restoration of Roles. |
| NISHIMOTO, A., OTAKA, Y., KASUGA, S., OTAKA, E., YAMAZAKI, K., USHIBA, J. & LIU, M., 2016. | Robotic assessment of upper limb function after proximal humeral fracture: Personal experience as a patient and occupational therapist. |
| O'BRIEN, R., 2017. | I want to work: A vocational rehabilitation case study following a brachial plexus injury. |
| QUACO, C., 2017. | Using a telehealth service delivery approach to working with an undergraduate student with a traumatic brain injury: A case study. |
| ROAN, C. & BELL, A., 2017. | Occupational Therapy in the Neonatal Intensive Care Unit for a Neonate with Perinatal Stroke: A Case Report. |
| ROSS, S.M., CATENA, M., TWARDZIK, E., HOSPODAR, C., COOK, E., AYYAGARI, A., INSKEEP, K., SLOANE, B., MACDONALD, M. & LOGAN, S.W., 2018. | Feasibility of a modified ride-on car intervention on play behaviors during an inclusive playgroup; Feasibility of a modified ride-on car intervention on play behaviors during an inclusive playgroup. |
| SUZUKI, Y., MIKAWA, T. & IKUTA, M., 2018. | Effects of mirror therapy on the reacquisition of motor imagery in patients with a hand orthopaedic injury. |
| SY, M.P. & OHSHIMA, N., 2019. | Utilising the Occupational Justice Health Questionnaire (OJHQ) with a Filipino drug surrenderee in occupational therapy practice: A case report. |
| TARADYS-RUSZKOWSKA, B. & KULINSKI, W., 2016. | Cognitive and emotional disturbances following a ischemic stroke within the rehabilitation process. |
| THOMAS, A., RAJA, K., NARAYANSETTI, N. & LAXMI, S., 2016. | Integrated rehabilitation for a case with head injury-Case report. |
| THWAITES, S., 2016. | Sensory integration principles in assessment of individuals with dementia |
| TORPIL, B., AKEL, S. & SAHIN, S., 2016. | Effect of sensory integration therapy on fine motor function in mitochondrial myopathy. |
| TUDOR, M., E.I. ICONARU, M. CIUCUREL, C. CIUCUREL & E. SOARE. , 2017. | A CASE STUDY OF OCCUPATIONAL THERAPY IN ACUTE MYOCARDIAL INFARCTION PATIENT |
| URUSHIDANI, N., KINOSHITA, S., OKAMOTO, T., TAMASHIRO, H. & ABO, M., 2018. | Low-Frequency rTMS and intensive occupational therapy improve upper limb motor function and cortical reorganization assessed by functional near-infrared spectroscopy in a subacute stroke patient. |
| UYESHIRO SIMON, A. & REEVES, L., 2016. | Lifestyle redesign occupational therapy for complex regional pain syndrome: A clinical case study. |
| VAIJANTI, R.J., THERAPY, R.S. & 2019, | Early Management of a Crush Hand injury Patient with Appropriate Splinting and Conventional Occupational Therapy: A Case Study. |
| VASSET, B., 2019. | Impact of working with community dogs for people with intellectual disability. |
| WALLACE, S.E., DONOSO BROWN, E.V., SAYLOR, A., LAPP, E. & ESKANDER, J., 2020. | Designing Occupational Therapy Home Programs for People With Aphasia: Aphasia-Friendly Modifications. Perspectives of the ASHA Special Interest Groups. |
| WILSON, L. & STABLEIN, R., 2017. | Role Differentiation in Occupational Therapy in Aotearoa New Zealand: Case Study of a Profession. |
| WIRT, Z., LOTZE, T. & NIEDZWECKI, C., 2016. | Acute inpatient rehabilitation management of spinal cord injury in pediatric neuromyelitis optica. |
| YOUNG, W., DAYA, M. & GOVENDER, P., 2019. | Functional outcome using early controlled active motion in rehabilitation of a replanted hand: A case report. |
| ZUZIAK, N.V. & THOMAS, N., 2019. | Thoracic myelopathy with paraplegia secondary to spontaneous spinal epidural abscess: A case study focusing on rehabilitation outcomes. |
| SAMAS, A. & NESTOR, K.K., 2018. | Poster 175: The Use of Craniosacral OMT and Occupational Therapy to Improve Upper Extremity Function in Congenital Brachial Plexus Palsy: A Case Report. |
| BALLOG, CARRANZA & LEE, 2020 | Environmental Impacts on the Occupations of Non-binary Individuals |
| GRADY-DOMINGUEZ ET AL, 2021 | Expectations and Assumptions: Examining the Influence of Staff Culture on a Novel School-Based Intervention to Enable Risky Play for Children with Disabilities |
| GRUHL, 2020 | Neoliberalism: Unpacking Limited Employment Success for Persons with SMI |
| HAZELTON ET AL, 2020 | The feasibility and effects of eye movement training for visual field loss afterstroke: a mixed methods study |
| HUOT ET AL, 2020 | Navigating Intersecting Forms of Oppression in the Search for Employment |
| MARSHALL ET AL, 2020 | Meaningful Activity and Boredom in the Transition from Homelessness:Two Narratives |
| PILLER ET AL, 2021 | Adapting Interventions for Occupational Therapy Practice: Application of the FRAME Coding Structure |
| SONDAY ET AL, 2020 | Case Study and Narrative Inquiry as Merged Methodologies: A Critical Narrative Perspective |
| ANISE, M. & PEREA, J., 2019. | Evidence-Based Interventions for Homonymous Hemianopia (HH): A Clinical Case Example. |
| BINKOWSKI, A., 2019. | Acute Flaccid Myelitis: Rehab Management for the Pediatric Patient. |
| DEAN, S. & BONDOC, S., 2016. | Goal attainment scaling and multi-component occupational therapy intervention for children with hemiplegia. |
| STEEL, E.J., BUCHANAN, R., LAYTON, N. & WILSON, E., 2017. | Currency and Competence of Occupational Therapists and Consumers with Rapidly Changing Technology. |
| TITCHENER, A., WALES, L. & DUNFORD, C., 2016. | A reflective case review: re-learning handwriting after a traumatic brain injury (TBI). |
| JUNG, N.-., 2019. | Effects of training of activities of daily livings on occupational performance and motivation in people with intellectual disability using day care center. |

*Exclusion Reason: Foreign language*

| Authors | Title |
| --- | --- |
| PSYCHIATRIE, C.V. & 2018, | Occupational therapy and eating disorders, a care case study. |

*Exclusion Reason: Context not occupational therapy practice*

| Authors | Title |
| --- | --- |
| ALDRICH, R.M., WHITE, N.A. & CONNERS, B.L., 2016. | Translating Occupational Justice Education Into Action. OTJR: Occupation, Participation and Health. 36(4), pp.227-233. |
| ALEKSANYAN, M., CHOUDHURY, M., OO, T.N. & SHIL, A.B., 2019. | Rehabilitation challenges in a patient with relapsing-remitting multiple sclerosis. |
| ASHISH, K., 2017. | Hirayama Disease -- A Case Study. |
| ATLER, K.E., BARNEY, L., MORAVEC, A., SAMPLE, P.L. & FRUHAUF, C.A., 2017. | The Daily Experiences of Pleasure, Productivity, and Restoration Profile: A case study. |
| BASTABLE, K., DADA, S. & UYS, C.J.E., 2016. | The Effect of a Non-Powered, Self-Initiated Mobility Program on the Engagement of Young Children with Severe Mobility Limitations in the South African Context. |
| BASU, A.P., PEARSE, J.E., FORSYTH, R. & BAKER, M., 2017. | Congenital mirror movements and unusual neurophysiology-is the reticulospinal tract to blame?. |
| BERG, L.A., JIRIKOWIC, T. & HAERLING, K., 2017. | Navigating the Hidden Curriculum of Higher Education for Postsecondary Students With Intellectual Disabilities. |
| BEYER, A., VANZALEN, L., BEDNARCZYK, M., STANKIEWICZ, C., SHAH, A., WILLIAMS, A. & PRITCHARD, K., 2019. | Using Data Science to Manage an Acute Care Rehabilitation Department. |
| BOES, C., 2016. | Importance assigned to cultural awareness, openness and responsiveness in occupational therapy education. |
| BONGADE, S.S., JAYWANT, S.S. & YERADKAR, R.S., 2018. | Effects of Specially Designed Early Self-Mobility Chair on Functional Independence in a Child with Myelomeningocele. |
| BRICE-LEDDY, L., PARK, D., BATEMAN, W., DRYSDALE, J., RATUSHNY, L., MUSSE, S. & NIXON, S.A., 2020. | Enabling Access to Rehabilitation in Acute Care: Exploring Physiotherapists' and Occupational Therapists' Perspectives on Patient Care When Assistants Become the Primary Therapy Providers. |
| BURTON, L.J. & CLARKE, D.J., 2017. | How do therapists select rehabilitation interventions? Selected findings from react, an ethnographic case study series in eight english stroke units. |
| BURTON, L.J. & TYSON, S., 2017. | How much of occupational therapists’ and physiotherapists’ time is spent in non-therapeutic activity? A qualitative case study in eight stroke units. |
| BUSETTO, L., KISELEV, J.Ö, LUIJKX, K. & VRIJHOEF, B.2016 | Implementation of integrated care at a German geriatric hospital: a case study. |
| CABATAN, M.C.C. & GRAJO, L.C., 2017. | Internationalization in an Occupational Therapy Curriculum: A Philippine-American Pilot Collaboration. |
| CAREY, H., 2017. | The positive impact of exercise for people with MND. Amyotrophic Lateral Sclerosis and Frontotemporal Degeneration. |
| CHADHA, C., DAHIYA, J., RAI, R. & CHUGH, P., 2017. | A Case Study- Effect of Constraint Induced Movement Therapy in Brain Tumor. Indian Journal of |
| CHAN, S.C.C., 2018. | Meaning making through occupational storytelling: Case study of a Chinese retiree. |
| CLOUSTON, T.J., 2019. | Pearls of wisdom: using the single case study or 'gem' to identify strategies for mediating stress and work-life imbalance in healthcare staff. |
| COLES, H.E., CARSON, S., DUKELOW, N., FLEEMAN, J.A., MAIER, S. & STAVISKY, C., 2014. | Integrative cognitive rehabilitation program: An innovative, multidisciplinary approach. |
| COLLINS, A.B., PRUITT, D.W., ROSE, J.B. & WILLIAMS, S.E., 2016. | Inpatient pain rehabilitation outcomes for adolescents with chronic pain and pain-associated disability secondary to sickle cell disease. |
| DANIEL, S., TRIGG, C., PARNELL, P., STENTON, C. & TAYLOR, M., 2018. | The implementation of a high intensity rehabilitation programme augmented by robotics and virtual reality. |
| DENZER-WEILER, C. & HREHA, K., 2018. | The use of animal-assisted therapy in combination with physical therapy in an inpatient rehabilitation facility: A case report. |
| DEPGUNDE, V. & KAMBLE, D.R., 2018. | Robotic intervention and occupational therapy rehabilitation in stroke: A case study. |
| DERAKHSHANRAD, S.A., PIVEN, E. & GHOOCHANI, B.Z., 2017. | Comparing the Cognitive Process of Circular Causality in Two Patients with Strokes through Qualitative Analysis. |
| FACCHIN, A., BESCHIN, N. & DAINI, R., 2017. | Rehabilitation of right (personal) neglect by prism adaptation: A case report. |
| FATOREHCHY, S., HOSSEINI, S.A., HAGHGOO, H. & HOSSEINZADEH, S., 2019. | The effect of gait enhancer mechanism on functional balance and endurance of walking in children with Cerebral Palsy. |
| FERREIRA, R.C., RIBEIRO, MARCO TULIO DE FREITAS, VARGAS-FERREIRA, F., SAMPAIO, A.A., PEREIRA, A.C.M., VARGAS, A.M.D., DE JESUS, R.M. & FERREIRA, E.F.E., 2018. | Assistive technologies for improving the oral hygiene of leprosy patients residing in a former leprosy colony in Betim, Minas Gerais, Brazil. |
| FLIPPIN, M., 2018. | Using father-mediated intervention to increase responsive parental behaviors and child communication in children with autism spectrum disorder: A pilot study. |
| FURUKAWA, T., KURIHARA, Y. & MASAKADO, Y., 2019. | The efficacy of ultrasound-guided selective botulinum toxin type a therapy for finger spasticity following stroke: A case report. |
| GAMIELDIEN, F. & VAN NIEKERK, L., 2017. | Street vending in South Africa: An entrepreneurial occupation. |
| GHUMARE, S.P., 2018. | Effect of home based occupational therapy rehabilitation on quality of life in transvers myelitis a single case study. |
| GROENEWOUD, H., SENHAJI, H., VAN 'T LEVEN, N. & DE LANGE, J., 2017. | Barriers and facilitators to improve the social network of home-dwelling people living with dementia and their family caregivers. |
| HARTE, D. & PATERSON, A., 2018. | The fastest field sport in the world: A case report on 3-dimensional printed hurling gloves to help prevent injury. |
| HEATWOLE SHANK, K.,S. & CUTCHIN, M.P., 2016. | Processes of developing 'community livability' in older age. |
| HESS-APRIL, L., SMITH, J. & DE JONGH, J., 2016. | Exploring occupational therapy graduates' conceptualisations of occupational justice in practice: Curriculum implications. |
| HOOPER, B., VERDONCK, M., AMSTERS, D., MYBURG, M. & ALLAN, E., 2018. | Smart-device environmental control systems: experiences of people with cervical spinal cord injuries. |
| HURST, H., 2016. | What has theory got to do with practice? Bridging the gap in integrated teams. |
| HUTTON, E. & SOAN, S., 2017. | ‘Lessons learned’ from introducing universal strategies designed to support the motor and functional skills of Reception and Year 1 children in a sample of primary schools in South East England. |
| INGERSOLL, B.R., WAINER, A.L., BERGER, N.I. & WALTON, K.M., 2017. | Efficacy of low intensity, therapist-implemented Project ImPACT for increasing social communication skills in young children with ASD. |
| JACKSON, Y. & SHARP, T., 2019. | PTA and OTA Interprofessional Education Collaboration: A Case Study. |
| KARP, P.&Therrell, James. 2019. | A case study to determine classroom and field educator perspectives on occupational therapy student readiness for transition to clinical practice. |
| KAWASHIMA, N., ISOGAI, M., MATSUHASHI, M., KOMACHI, M., IKEBE, H., KUMON, A., MIYASHITA, K., SATO, A. & HASEGAWA, K., 2018. | The effectiveness of boxing exercise in elderly patients including Parkinson's disease patients |
| KENYON, L.K., FARRIS, J.P., GALLAGHER, C., HAMMOND, L., WEBSTER, L.M. & ALDRICH, N.J., 2017. | Power Mobility Training for Young Children with Multiple, Severe Impairments: A Case Series. |
| KO, K., HA, G. & KANG, S., 2017. | Effects of daily living occupational therapy and resistance exercise on the activities of daily living and muscular fitness in Guillain-Barré syndrome: a case study. |
| KURODA, M., NAKAGAWA, S., MUTSUZAKI, H., MATAKI, Y., YOSHIKAWA, K., TAKAHASHI, K., NAKAYAMA, T. & IWASAKI, N., 2020. | Robot-assisted gait training using a very small-sized Hybrid Assistive Limb for pediatric cerebral palsy: A case report. |
| LAING, K., 2019. | How do occupational therapists and classroom teachers work across professional boundaries to support students who have profound intellectual and multiple disabilities?. |
| LEVINE, D., CROSS, C., MATTHEWS, J., MCDONALD, S. & FELL, N., 2019. | The Effect of Animal Assisted Therapy on Participation in Rehabilitation in a Patient Post-Stroke. |
| LIU, C.-. & YU, N.-., 2019. | Setting Therapeutic Goals for a Child With Cerebral Visual Impairment: A Case Study. |
| LONGPRE & POLO, 2020 | A Personal Perspective on Daily Occupations to Counteract Cancer Related Fatigue: A Case Study |
| MCKINSTRY, C., IACONO, T., KENNY, A., HANNON, J. & KNIGHT, K., 2020. | Applying a digital literacy framework and mapping tool to an occupational therapy curriculum. |
| MERINO, GISELLE S. A. D., R.F. PICHLER, S. DOMENECH, Z. RECH, E.A.D. MERINO & F. REBELO. , 2018. | Design of Assistive Devices and Occupational Therapy: Case Study in a Brazilian Psychatric Hospital |
| MISHRA, A.N., 2018. | Hand based functional training for hemiparetic patient with in-hand manipulation deficit using cognitive approach a single case study. |
| NED, L., CLOETE, L. & MJI, G., 2017. | The experiences and challenges faced by rehabilitation community service therapists within the South African Primary Healthcare health system. |
| PIVEN, E.F. & DERAKHSHANRAD, S.A., 2017. | A case study demonstrating reduction of aggressive client behaviors using the neuro-occupation model: Addressing professional burnout through nonlinear thinking; A case study demonstrating reduction of aggressive client behaviors using the neuro-occupation model: Addressing professional burnout through nonlinear thinking. |
| PROFFITT, R. & HENDERSON, W., 2016. | LSVT-BIG in stroke: A case study. Archives of Physical Medicine and Rehabilitation. |
| REBEIRO GRUHL, 2017 | Becoming visible: Exploring the meaning of busking for a person with mental illness |
| RICCI, FLÁVIA PESSONI F. M., MCKEE, P., ZAMPAR, A.C., GRILLO SEMEDO, A.C., PEREIRA SANTIAGO, P.R. & FONSECA, MARISA DE CÁSSIA REGISTRO, 2020. | Enhancing function after radial nerve injury with a high-profile orthosis and a bio-occupational orthotic framework. |
| ROGELJ, P. & ZAJC, D., 2019. | Effectiveness of robotics or sensory-supported training in improving upper extremity functions among people with multiple sclerosis - Case study. |
| SAKETA, J. & SUBBIAH, K., 2016. | A Treatment based Classification Approach to Mechanical Lesion of Shoulder for Conservative Management and Improvement in Clinical Outcomes. |
| SCHUMACHER, K., KOCH, K. & ESHRAGHI, N., 2019. | The role of burn rehabilitation on burn patients receiving extracorporeal membrane oxygenation. |
| SMITH, A.J., CAMPBELL, R.W., HARRISON, P.K. & HARRISON, D.W., 2016. | Functional cerebral space theory: Towards an integration of theory and mechanisms of left hemineglect, anosognosia, and anosodiaphoria. |
| SÖDERQVIST, H., KAJSA, E., AHLSTRÖM, B.H. & WENTZ, E., 2017. | The caregivers’ perspectives of burden before and after an internet-based intervention of young persons with ADHD or autism spectrum disorder. |
| SORENSEN, L. & MANUM, G., 2019. | A single-subject study of robotic upper limb training in the subacute phase for four persons with cervical spinal cord injury. |
| SPINELLI, L., LAVELLE, M. & FELD-GLAZMAN, R., 2017. | Treating facial asymmetry using kinesio tape to maximize function and improve well-being post-stroke: A case study. |
| STERMAN, J., 2018 | Is Play a Choice on the School Playground for Children With Disabilities? |
| STRONG, D.M., 2018. | Supporting the "Casa Lar" social educator: a case study of a consultation using intercultural knowledge translation. |
| TAYLOR, T., HABERLIN, A. & HABERLIN, J., 2019. | Treatment of avoidant/restrictive food intake disorder for a teenager with typical development within the home setting. |
| TSERTSIDIS, A., 2020. | Challenges in the provision of digital technologies to elderly with dementia to support ageing in place: a case study of a Swedish municipality. |
| VERNESE, L.F., CARA, J., BOUFFARD, K. & TYNER, T., 2017. | Effective treatment of axial neck pain and cervicogenic headaches secondary to ankylosing spondylitis through a comprehensive, multidisciplinary pain management program: A case report. |
| VIKAS, C.K. & DIVYA PARASHAR, R.A., 2019. | Effect of Early Rehabilitation in a Subject with Conversion Disorder Presented as Paraplegia: A Case Study |
| VILLANUEVA, C.G.&Davis, Kay. 2016. | An exploratory case study about interprofessional simulation-based learning for a team of health care educators. |
| VINING, R.D., GOSSELIN, D.M., THURMOND, J., CASE, K. & BRUCH, F.R., 2017. | Interdisciplinary rehabilitation for a patient with incomplete cervical spinal cord injury and multimorbidity: A case report. |
| VIVAS, L.L.Y., GOLD, W.L., MANDELL, D.M. & WU, P.E., 2016. | An 82-year-old man with ataxia and dysarthria. |
| VOGT, C., 2017. | The risk of misdiagnosing posture weakness as hyperactivity in ADHD: a case study. |
| WARYOBA, D.R., L. DEMI & A. FATULA. , 2016. | A Case Study of Interprofessional Collaboration Between Engineering and Health Sciences Students at Penn State DuBois |
| WHITE, B.P., BRINKMAN, A., KRESGE, B.P. & COUTURE, L., 2018. | Quality of Life, Stress Perception, and Quality of Social Networks in Persons Living with Brain Injury: An Exploration of the Effectiveness of a Community-Based Program. |
| WILSON, C.M., MITCHELL, C.L. & HEBERT, K.M., 2017. | Cerebellar Stroke Occupational Therapy and Physical Therapy Management from Intensive Care Unit to Outpatient: A Case Report. db=cmedm&AN=29468104&authtype=sso&custid=s8849815&site=ehost-live&scope=site"]. |
| YAM, A., RICKARDS, T., PAWLOWSKI, C.A., HARRIS, O., KARANDIKAR, N. & YUTSIS, M.V., 2016. | Interdisciplinary rehabilitation approach for functional neurological symptom (conversion) disorder: A case study. |
| ZIDIANAKIS, E., ANTONA, M. & STEPHANIDIS, C., 2018. | ACTIVITY ANALYSIS (ACTA): EMPOWERING SMART GAME DESIGN WITH A GENERAL PURPOSE FSM DESCRIPTION LANGUAGE. |
| AVERY ET AL 2021 | Comparison of Trigger Finger Orthotic Wearing Schedules: A Feasibility Study |
| COLES & ELLIOTT, 2020 | From reflection to action: a case study of an art psychotherapy group which drew on occupational therapy perspectives |
| DONODO BROWN ET AL, 2020 | Effects of Preferred Music Listening on Adherence to Upper Extremity Home Programs |
| ELLIS ET AL, 2020 | Sexuality and Intimacy Rehabilitation for the Military Population: Case Series |
| FRIAS 2020 | Exploring the Use of Mobile Electroencephalogram (EEG) in Driving Assessment:A Case Study |
| GEE, 2021 | Weighted Blankets and Sleep Quality in Children with Autism Spectrum Disorders: A Single-Subject Design |
| JAYWANT ET AL, 2020 | Feasibility and acceptability of the multicontext approach for individuals with acquired brain injury in acute inpatient rehabilitation: A single case series |
| APABLAZA, M.2018. | Inclusion in education, occupational marginalization and apartheid: An analysis of Chilean education policies. |
| AUBIN, G., BÉLIVEAU, M. & KLINGER, E., 2018. | An exploration of the ecological validity of the Virtual Action Planning-Supermarket (VAP-S) with people with schizophrenia. |
| CARANDANG, K. & PYATAK, E.A., 2018. | Analyzing occupational challenges through the lens of body and biography. |
| CATALANO, A., 2017. | Occupational Profiles of At-Risk Youth: A Multi-Case Study. Occupational Profiles of at-Risk Youth: A Multi-Case Study. |
| CHEN, Z., 2019. | A novel staged wrist sensorimotor rehabilitation program for a patient with triangular fibrocartilage complex injury: A case report. |
| COSBEY, J. & MULDOON, D., 2017. | EAT-UP™ Family-Centered Feeding Intervention to Promote Food Acceptance and Decrease Challenging Behaviors: A Single-Case Experimental Design Replicated Across Three Families of Children with Autism Spectrum Disorder. |
| BRÍD DUNNE, B.,B.A., PETTIGREW, J. & ROBINSON, K., 2017. | From the wretched laundry to a lovely solarium: the history of the therapeutic use of occupation in Ireland, 1863-1970. |
| FRAGA, E., DA SILVA DITTZ, E. & GUIMARÃES MACHADO, L., 2019. | The construction of maternal co-occupation in the Neonatal Intensive Care Unit. |
| HART, E.C. & HEATWOLE SHANK, K. PARTICIPATING AT THE MALL: POSSIBILITIES AND TENSIONS THAT SHAPE OLDER ADULTS' OCCUPATIONS. , 2016. | Participating at the Mall: Possibilities and Tensions that Shape Older Adults' Occupations |
| HOUGAARD, B.I.&H. KNOCHE. , 2019. | Telling the Story Right: How Therapists Aid Stroke Patients Interpret Personal Visualized Game Performance Data |
| HUOT, S. & VERONIS, L., 2018. | Examining the role of minority community spaces for enabling migrants’ performance of intersectional identities through occupation. |
| KRENZER, M.L.M., 2019. | FACULTY OF HEALTH SCIENCES DEPARTMENT OF HEALTH AND REHABILITATION SCIENCES DIVISION OF OCCUPATIONAL THERAPY. |
| KHATRI, R., LOGAN, P., KAY, A. & LEHNER, A., 2016. | Does splinting the upper limb improve the quality of life and functional independence of stroke survivors? |
| RAMUGONDO, E., 2019. | Everyday enactments of humanity affirmations in post 1994 apartheid South Africa: a phronetic case study of being human as occupation and health. |
| LIU, C.-., 2017. | The effects of cognitive orientation to daily occupational performance approach for motor coordination disorder. |
| MORGAN-BROWN, M. & BRANGAN, J., 2016. | Capturing Interactive Occupation and Social Engagement in a Residential Dementia and Mental Health Setting Using Quantitative and Narrative Data. |
| MORRIS, K., COX, D.L. & WARD, K., 2016. | Exploring stories of occupational engagement in a regional secure unit. |
| RIPAT, J., BORISOFF, J.F., GRANT, L.E. & CHAN, F.H.N., 2018. | Patterns of community participation across the seasons: A year-long case study of three Canadian wheelchair users. |
| SANDERS, M.J., 2018. | Older Manufacturing Workers and Adaptation to Age-Related Changes. |
| SOKMEN, Y.C. & WATTERS, A., 2016. | Emotion regulation with mindful arts activities using a personalized self-soothing kit; Emotion regulation with mindful arts activities using a personalized self-soothing kit. |
| STÅHL, A. & MÅNSSON LEXELL, E., 2018. | Facilitators for travelling with local public transport among people with mild cognitive limitations after stroke. |
| STARK, S., LORENZO, T. & LANDMAN, S., 2016. | Shaped by place: Environmental influences on the participation of young cyclists from disadvantaged communities in professional cycling. |
| STERMAN, J.J., NAUGHTON, G.A., BUNDY, A.C., FROUDE, E. & VILLENEUVE, M.A., 2019. | Mothers supporting play as a choice for children with disabilities within a culturally and linguistically diverse community. |
| STERMAN, J.J., NAUGHTON, G.A., BUNDY, A.C., FROUDE, E. & VILLENEUVE, M.A., 2019. | Planning for outdoor play: Government and family decision-making. |
| SWANEPOEL, A., THERAPY, S.M. & 2018, | The roles and the effect of role expectations on the academic performance of first year occupational therapy students: a University of the Free State case study. |
| TOMSONE, S., HAAK, M. & LÖFQVIST, C., 2016. | Experiences of mobility device use over time: A multiple case study among very old Latvian women. |
| VOUSDEN, B., WILKES‐GILLAN, S., CORDIER, R. & FROUDE, E., 2019. | The play skills of children with high‐functioning autism spectrum disorder in peer‐to‐peer interactions with their classmates: A multiple case study design. |
| WARREN, K., HUOT, S., MAGALHAES, L. & EVANS, M., 2016. | Exploring the Daily Lives of People on Methadone Maintenance Treatment: An Occupational Perspective. |
| WASHINGTON, S.E., 2020. | Fostering empowerment through occupation: An overview of an urban school-based parenting program. |
| WOMACK, J.L., ISAKSSON, G. & LILJA, M., 2016. | Care partner dyad strategies to support participation in community mobility. |
| WONG, C 2018. | Meaningful Engagement in a Green House Nursing Home |
| L., W., 2017. | A developmental perspective informs a framework of self-awareness following traumatic brain injury in childhood |
| ASHLEY ET AL, 2019 | The home environments and occupational engagement of people with intellectual disabilities in supported living |
| CAMERON ET AL | Return-to-work support for employees with mental health problems: Identifying and responding to key challenges of sick leave |
