# Supplementary material for: Exploring the contribution of case study research to the evidence base for occupational therapy: a scoping review
Source: Syst Rev. 2023 Jul 31;12:132. doi: 10.1186/s13643-023-02292-4 (PMC10388505; doi:10.1186/s13643-023-02292-4)
Supplement: Supplementary file 8 — Additional file 8. Heat map contrasting interventions and practice contexts. Numbers and shading represent number of studies. [file 13643_2023_2292_MOESM8_ESM.docx]

| **Practice setting context / Intervention categories** | **Community** | **Inpatient** | **Not reported** | **Outpatient clinic** | **School** | **Third sector** | **Grand Total** |
| --- | --- | --- | --- | --- | --- | --- | --- |
| **Animal assisted** | 2 |  |  |  |  |  | 2 |
| **Assistive device and equipment** | 7 | 1 | 1 |  |  |  | 9 |
| **Broader practice focus** | 7 | 1 | 1 |  |  | 2 | 11 |
| **Cognitive Orientation to daily Occupational Performance (CO-OP)** | 5 |  | 1 |  |  |  | 6 |
| **Compression bandaging** |  | 2 |  |  |  |  | 2 |
| **Family based interventions** | 3 |  |  | 1 |  |  | 4 |
| **Feeding** |  |  |  | 1 |  |  | 1 |
| **Functional electrical stimulation / Task specific training** | 1 | 1 |  | 1 |  |  | 3 |
| **Gaming** | 1 |  | 2 | 1 |  |  | 4 |
| **Gardening** | 1 |  |  |  |  |  | 1 |
| **Lee Silverman Voice Technique BIG** | 1 |  | 1 | 1 |  |  | 3 |
| **Mind body / psychomotor interventions** |  | 1 | 1 | 1 |  |  | 3 |
| **Motor imagery** |  |  |  | 1 |  |  | 1 |
| **Non-specific individualized occupational therapy** | 4 |  |  |  |  |  | 4 |
| **Occupational adaptation mentoring programme** | 1 |  |  |  |  |  | 1 |
| **Occupational Performance Coaching** | 1 |  | 1 |  | 1 |  | 3 |
| **Orthotics and prosthetics** |  |  |  | 1 |  |  | 1 |
| **Play** | 2 | 1 |  | 1 |  |  | 4 |
| **Reablement** | 1 |  |  |  |  |  | 1 |
| **Self-management** | 1 |  | 1 |  |  |  | 2 |
| **Sexual health promotion** | 1 |  |  |  |  |  | 1 |
| **Somatosensory or sensory based** | 4 | 1 | 2 | 2 | 1 |  | 10 |
| **Theatre** |  |  |  |  |  | 1 | 1 |
| **Vision based** | 1 |  |  |  |  |  | 1 |
| **Vocational** | 2 |  |  | 1 |  |  | 3 |
| **Wheeled mobility** | 2 |  |  |  |  |  | 2 |
| **Grand Total** | 48 | 8 | 11 | 12 | 2 | 3 | 84 |

|  |  |  |  |  |  |
| --- | --- | --- | --- | --- | --- |

***0 1 2-4 5-7 8-11 12+***
