# Supplementary material for: Exploring the contribution of case study research to the evidence base for occupational therapy: a scoping review
Source: Syst Rev. 2023 Jul 31;12:132. doi: 10.1186/s13643-023-02292-4 (PMC10388505; doi:10.1186/s13643-023-02292-4)
Supplement: Supplementary file 9 — Additional file 9. Heat map contrasting population characteristics of age and diagnosis categories. Numbers and shading represent number of studies. [file 13643_2023_2292_MOESM9_ESM.docx]

| **Population Diagnosis / Age** | **Brain injury** | **Cancer** | **Concussion** | **Dementia** | **Mental ill-health** | **Multiple Sclerosis** | **Neuro-developmental disorder** | **Non-specific** | **Parkinson Disease** | **Physical disability** | **Spinal injury** | **Stroke** | **Substance misuse** | **Visual impairment** | **Grand Total** |
| --- | --- | --- | --- | --- | --- | --- | --- | --- | --- | --- | --- | --- | --- | --- | --- |
| **Adult (18+)** | 3 |  |  |  | 7 | 1 | 3 | 1 | 1 | 1 | 1 | 9 |  |  | 27 |
| **Children (0–17)** |  | 1 |  |  | 1 |  | 18 | 3 |  | 1 |  |  |  |  | 24 |
| **Mixed ages groups** |  |  | 1 |  |  |  | 11 | 3 |  |  |  | 3 | 1 |  | 21 |
| **Not reported** |  |  |  |  | 1 |  |  | 1 |  |  |  | 1 |  |  | 3 |
| **Not applicable** |  |  |  |  |  |  |  | 3 |  |  |  |  |  |  | 3 |
| **Older adult (65+)** |  |  |  | 1 |  |  |  | 2 | 1 |  |  | 1 |  | 1 | 6 |
| **Grand total** | **3** | **1** | **1** | **1** | **9** | **1** | **32** | **13** | **2** | **2** | **1** | **14** | **1** | **1** | **84** |

|  |  |  |  |  |  |
| --- | --- | --- | --- | --- | --- |

***0 1 2-4 5-7 8-11 12+***
